# Identifying Bioaccumulative Halogenated Organic Compounds Using a Nontargeted Analytical Approach: Seabirds as Sentinels

**DOI:** 10.1371/journal.pone.0127205

**Published:** 2015-05-28

**Authors:** Christopher J. Millow, Susan A. Mackintosh, Rebecca L. Lewison, Nathan G. Dodder, Eunha Hoh

**Affiliations:** 1 Ecology Program Area, Department of Biology, San Diego State University, San Diego, California, United States of America; 2 Graduate School of Public Health, San Diego State University, San Diego, California, United States of America; 3 San Diego State University Research Foundation, San Diego, California, United States of America; 4 Southern California Coastal Water Research Project Authority, Costa Mesa, California, United States of America; Moffitt Cancer Center, UNITED STATES

## Abstract

Persistent organic pollutants (POPs) are typically monitored via targeted mass spectrometry, which potentially identifies only a fraction of the contaminants actually present in environmental samples. With new anthropogenic compounds continuously introduced to the environment, novel and proactive approaches that provide a comprehensive alternative to targeted methods are needed in order to more completely characterize the diversity of known and unknown compounds likely to cause adverse effects. Nontargeted mass spectrometry attempts to extensively screen for compounds, providing a feasible approach for identifying contaminants that warrant future monitoring. We employed a nontargeted analytical method using comprehensive two-dimensional gas chromatography coupled to time-of-flight mass spectrometry (GC×GC/TOF-MS) to characterize halogenated organic compounds (HOCs) in California Black skimmer (*Rynchops niger*) eggs. Our study identified 111 HOCs; 84 of these compounds were regularly detected via targeted approaches, while 27 were classified as typically unmonitored or unknown. Typically unmonitored compounds of note in bird eggs included tris(4-chlorophenyl)methane (TCPM), tris(4-chlorophenyl)methanol (TCPMOH), triclosan, permethrin, heptachloro-1'-methyl-1,2'-bipyrrole (MBP), as well as four halogenated unknown compounds that could not be identified through database searching or the literature. The presence of these compounds in Black skimmer eggs suggests they are persistent, bioaccumulative, potentially biomagnifying, and maternally transferring. Our results highlight the utility and importance of employing nontargeted analytical tools to assess true contaminant burdens in organisms, as well as to demonstrate the value in using environmental sentinels to proactively identify novel contaminants.

## Introduction

Declining populations of many marine species is a growing concern [1, 2] and exposure to toxic and bioaccumulative compounds has been implicated as one causal factor [3], especially among seabirds [4]. Persistent organic pollutants (POPs) including polybrominated diphenyl ethers (PBDEs), polychlorinated biphenyls (PCBs), and dichlorodiphenyltrichloroethanes (DDTs) have been introduced into waterways via urban runoff and anthropogenic activities. The presence of these compounds has been shown to cause reproductive and physiological impairments in wildlife, including avifauna [5–8]. To date, evaluation of chemical loads in marine wildlife has focused primarily on known compounds using targeted analytical approaches to detect legacy contaminants such as PCBs and DDTs. However, the current list of typically monitored organic pollutants only represents a fraction of the number of compounds potentially present in wildlife. Unmonitored and/or unknown compounds, with the potential to cause adverse biological impacts, are largely ignored or unrecognized due to economic or analytical limitations [9]. Thus, nontargeted contaminant screening of marine species broadens the scope of analysis to include both typically monitored and unmonitored contaminants, providing a more thorough understanding of their impacts on sensitive populations.

To address the challenges of screening for multiple classes of known and unknown compounds, novel approaches that provide a proactive and comprehensive alternative to targeted mass spectrometry are needed. In this study, a nontargeted analytical method, developed by Hoh et al. (2009; 2012), was applied to analyze halogenated organic compounds (HOCs) in seabird eggs. Comprehensive two-dimensional gas chromatography coupled to time-of-flight mass spectrometry (GC×GC/TOF-MS) was used to characterize persistent, bioaccumulative, and potentially toxic HOCs in the samples. This method has been used to successfully analyze compounds in Atlantic common dolphin blubber and fish oil [10, 11].

While various marine species have been used as environmental sentinels, the unique utility of piscivorous seabirds has been documented consistently in the literature [4, 12]. Piscivorous seabirds typically have higher contaminant levels than seabirds feeding on plankton or other lower trophic level prey, and additionally have lower variance in organochlorine concentrations than do fish or marine mammals. Therefore, a relatively small sample size of seabird tissues (i.e. eggs or blood) can be collected from centralized colonies to provide sufficient statistical power with limited environmental impact and low sampling costs [13]. Lipid-rich seabird eggs provide an ideal matrix to measure lipophilic contaminants [14], where concentrations reflect biomagnification and maternal transfer.

Nontargeted analytical approaches may be particularly valuable for monitoring highly impacted, urbanized sites due to the potential influx of unrecognized and/or unknown anthropogenic compounds. Here, we demonstrate the utility of the nontargeted analytical approach, using GC×GC/TOF-MS, in identifying HOCs in a piscivorous seabird, the Black skimmer (*Rynchops niger*), inhabiting an urbanized coastal area of San Diego Bay, CA. Despite relatively stable colony sizes, nationwide reproductive output of skimmers has remained low or declined in the past two decades [15–17]. Due to these low numbers, Black skimmers are considered a “Species of Special Concern” and a conservation priority in California. In other states, they are listed as endangered, e.g., in New Jersey where the population has declined over the last 20 years [18].

In 2001, a coast-wide survey revealed that fish in the San Diego Bay had some of the highest levels of PBDEs in California [19]. In San Diego Bay, PCBs, DDTs, and PBDEs in skimmers were reported in both greater total number of compounds and higher levels than similar co-occurring species, and at levels thought to affect reproduction [20]. With high contaminant loads, skimmers serve as sensitive indicators of coastal health and of contaminant threats to piscivorous birds and other high trophic-level consumers.

Our results highlight the importance of employing comprehensive analytical tools to assess true contaminant burdens in organisms, as well as demonstrate the value of using environmental sentinels, like seabirds, to proactively identify both emerging and previously unknown contaminants.

## Materials and Methods

### Ethics statement

All sample collection and animal handling methods were approved by the Institutional Animal Care and Use Committee (IACUC) at San Diego State University, the California Department of Fish and Game (Permit: SC-11656), the US Fish and Wildlife Service (Permit: MB42753A-0), and the US Geological Survey Bird Banding Laboratory (Permit: 22873).

### Study location

San Diego Bay (SDB) is a 36 km^2^ highly urbanized natural harbor and deepwater port impacted by runoff, international commercial shipping, Naval activities, and recreational boating. It is located near the US-Mexico border and is the terminus of three watersheds comprising approximately one million residents. Inflow occurs from the Sweetwater and Otay Rivers located at the south end of SDB. SDB has an extensive history of pollution levels exceeding national water quality standards [21] and fauna toxicity thresholds [22, 23]. Remnant coastal wetlands within the Bay—as well as the largest contiguous mudflat in southern California—provide critical habitat for over 300 bird species including a mixed migratory and resident population of Black skimmers, the focal species of this study.

Field research and egg sample collection was conducted in the South San Diego Bay unit of the San Diego Bay National Wildlife Refuge (32°35’56.81” N, 117°6’11.32” W). Within the refuge, the sole breeding colony of Black skimmers is centered at the South Bay Salt Works, an active salt extraction facility where dense congregations of seabirds nest on kilometers of raised levees.

### Study species: Black skimmers

Black skimmers are long-lived (upwards of 20 years; we banded one nesting female in 2011 that was 21 years old), colonial-nesting, piscivorous seabirds found throughout the coastal Americas, and are year-round residents of San Diego County. They lay one to four eggs in a shallow nest on the ground. Their unique foraging method restricts them to surface waters, making it advantageous to feed in shallow wetland margins that may also present higher toxin exposure [24]. Within California, only four major breeding colonies—which experience frequent population mixing—exist and are confined to the southern third of the state: Bolsa Chica, Seal Beach, Salton Sea, and San Diego Bay-Salt Works. San Diego Bay hosts the largest of the four colonies, which accounts for nearly half of the California population [25].

### Sample collection and preparation

Black skimmer egg samples were collected from the Salt Works colony in June, July, and August 2011. All eggs were opportunistically marked to document laying order. Four eggs representing different nests and weeks within the breeding season were randomly selected for analysis and were labeled as egg A, B, C, and D. Eggs were frozen at -20°C within 6 hours of collection. Eggs were later thawed and contents transferred to glass beakers that were previously baked at 450°C for 6 hours. Thawed egg contents were homogenized for 1 minute with an immersion blender (IKA Works Inc, Wilmington, NC, USA) cleaned between each sample with distilled water, acetone, and hexane. Homogenized samples were stored at -20°C until further analysis.

### Sample preparation for chemical analysis

Further information on exact sample protocol and reference standards is available in S2 File. Briefly, for each sample, five grams of egg homogenate was transferred to a baked 50 mL glass Pyrex centrifuge tube and spiked with known amounts of internal standards. Prepared simultaneously was a laboratory procedural blank sample (5g nano-pure LCMS grade water). One mL of 6N hydrochloric acid (HCl) and 5 mL of isopropanol were added to each sample to denature proteins. Samples then underwent a liquid-liquid extraction with 5 mL of 1:4 (v:v) dichloromethane (DCM):hexane followed by centrifugation for 15 minutes at 3000 rpm and 20°C (repeated twice). One mL of the sample extract was then transferred to an aluminum pan for gravimetric lipid analysis. The remaining extract from each sample was placed in a Zymark TurboVap (Caliper Life Sciences, Hopkinton, MA, USA), and concentrated to approximately 2 mL with nitrogen and a water-bath held at 40°C.

Extracts were further cleaned via gel permeation chromatography (GPC; J2 Scientific, Columbia, MO, USA), in order to remove lipids, followed by silica-based solid phase extraction (SPE; Environ-Clean Silica 1000 mg/6 mL Glass, UCT, Bristol, PA, USA). Using a vacuum manifold, extracts were eluted with 4 mL of hexane followed by 4 mL of 1:9 (v:v) hexane:DCM. Samples were then evaporated under nitrogen, transferred to a 2 mL amber vial, and spiked with known amounts of recovery standards. The final sample volume was 100 μL prior to instrumental analysis.

### Instrumentation for chemical analysis

Egg samples were analyzed using a Pegasus 4D GC×GC/TOF-MS (LECO, St. Joseph, MI, USA). One μL of the final extract was injected with splitless mode and with research grade helium (99.995% Airgas Wes El Cajon, CA USA) as the carrier gas. The instrument parameters are summarized in S1 Table.

### Compound identification and quantification

Initial compound identification was assisted by the LECO ChromaToF optimized for Pegasus 4D (version 4.43.3.0) software. The 2011 National Institute of Standards and Technology (NIST) Mass Spectral Library was used for compound peak identification, by automated matching of library mass spectra to the experimental mass spectra. After the automatic peak identification was conducted, manual review was necessary for verification and final determination of compounds of interest. Selection criterion for determining ‘Compounds of Interest’ was as follows: chromatographic peaks at S/N ≥ 50 were selected; next, compounds had to contain at least one halogen (i.e., chlorine; bromine) which was identified by characteristic halogen isotopic clustering in the mass spectrum. Compounds of interest within each of the four egg samples were then cross-referenced with each other to compile a master list of compounds of interest. Compounds of interest were retained if their peak areas were at least three times larger than their corresponding peaks in the lab blank.

Identifications fell within the following categories [10], with the category names in brackets: (1) the experimental mass spectrum and retention times were matched to those of a reference standard analyzed under the same conditions [authentic MS RT]; (2) the experimental mass spectrum, but not the retention times, was matched to a reference standard, indicating the experimental spectrum is that of an isomer [authentic MS]; (3) the experimental mass spectrum was matched to one within the NIST Electron Ionization Mass Spectral Library [reference database MS]; (4) the experimental spectrum was matched to one found in literature [literature MS]; (5) the experimental mass spectrum was identified as potentially belonging to a class of congeners on the basis of comparison to that of a reference standard within the same class of congeners [manual-congener group]; (6) a presumptive identification was made by manual interpretation of the experimental spectrum [manual]; (7) the experimental spectrum was identified as belonging to a halogenated compound, but the chemical structure could not be further identified [unknown] [10]. Relative peak response was calculated as a proxy for compound concentration by dividing the peak area of each compound by the peak area of the internal standard that most closely resembled the particular compound’s retention time: compounds with first dimension retention times (rt1D) of 0–1900 seconds were divided by the peak area of internal standard PCB65; compounds with rt1D of 1901–2199 seconds were divided by the peak area of internal standard PCB155; compounds with rt1D of 2200+ seconds were divided by the peak area of internal standard PCB204. Peak areas of all PBDE compounds were divided by the peak area of PBDE internal standard FBDE-4001S (4'-fluoro-2,3',4,6-tetrabromodiphenyl ether). Normalization of sample weights with regards to lipid content was not necessary, as lipid content was consistent across each of the four samples.

## Results and Discussion

### Nontargeted analytical approach

The nontargeted method we present here employed a halogenomic approach, i.e., the mass spectrum of every compound detected within a sample was visually examined for characteristic halogen isotope clusters (containing chlorine, bromine, or iodine), and was compared to a mass spectral library of known compounds. This screening method potentially captures a wider array of halogenated contaminants compared to targeted monitoring.

Each of four Black skimmer eggs yielded thousands of chromatographic peaks (S/N ≥ 50) indicating the maximum number of potential compounds of interest per sample (Egg A: n = 9998; Egg B: n = 7306; Egg C: n = 8544; Egg D: n = 5449). From these peaks, we identified a total of 111 unique halogenated organic compounds (HOCs) of anthropogenic, natural, and unknown origin; 61 percent of compounds were detected in all four eggs (n = 68), and 80 percent (n = 89) were detected in at least three eggs. Of the 111 compounds of interest, 84 compounds were typically monitored and 27 compounds were typically unmonitored. Among the typically unmonitored 27 compounds, 4 compounds contained halogens, but a complete chemical structure could not be identified and they were classified as unknown. S2 File contains the mass spectral library including the compound name, chemical structure, compound class, identification confidence, source, retention times, and mass fragment identities for all typically unmonitored compounds (n = 27). The library is also available as the R package SpecLibBlackSkimmerEgg2014 at http://OrgMassSpec.github.io/. The use and structure of the mass spectral library package was described previously [10].

**Fig 1:**
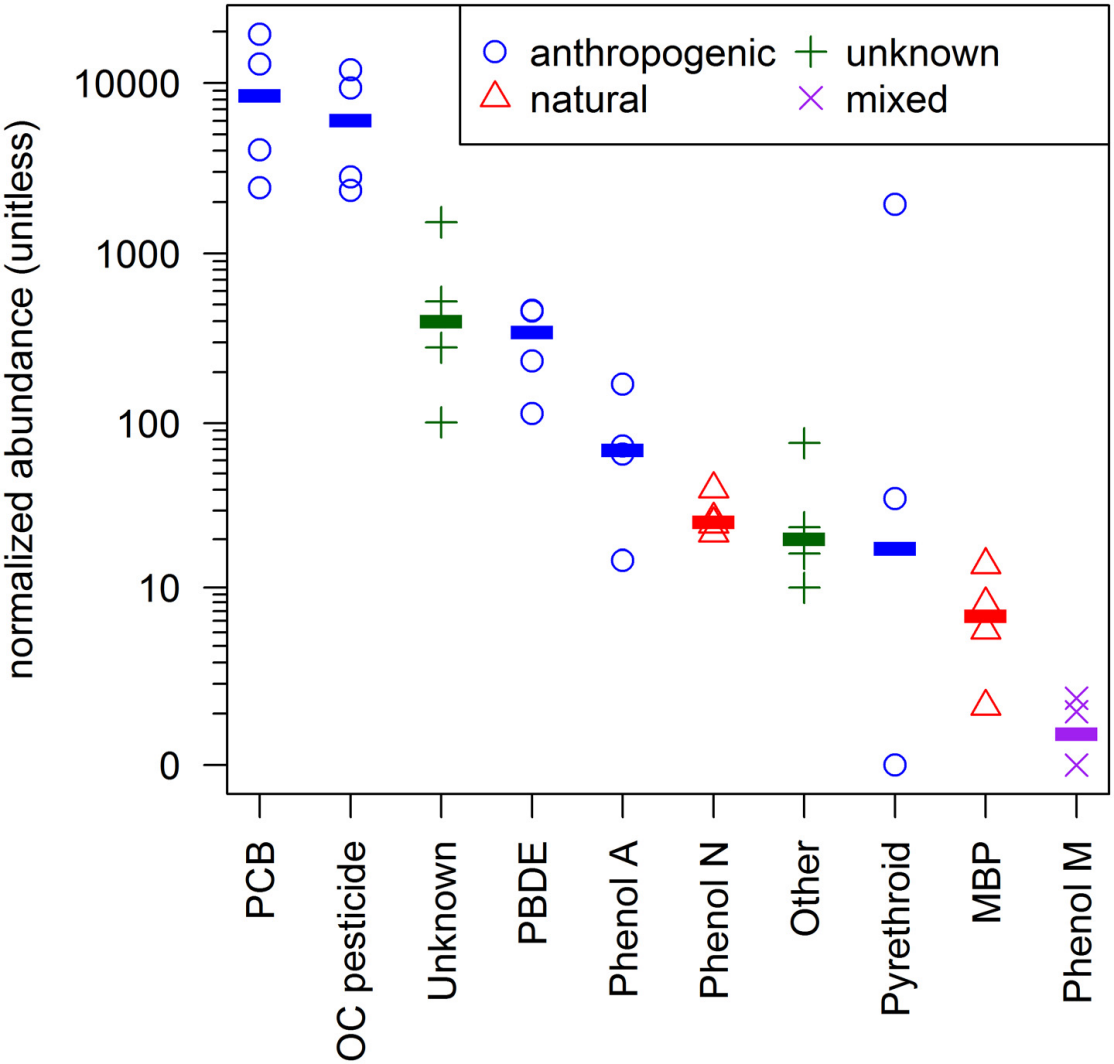
Relative Abundance of Compound Classes. Each point represents detection in a single egg sample (n = 4). The abundance is the sum total of the normalized peak areas for all compounds in the class, in each sample. The line is the median abundance for the compound class. The source of each compound class is indicated in the legend. Non-detects are shown with a value of zero (multiple non-detects overlap).

Each of the 111 compounds were assigned to a compound class (8 total), listed in order of highest to lowest abundance: polychlorinated biphenyls (PCBs; n = 65), organochlorine pesticides (OCs; n = 20), pyrethroids (n = 3), unknowns (n = 4), polybrominated diphenyl ethers (PBDEs; n = 8), phenols (n = 7; 5 anthropogenic and 2 natural), other halogenated compounds (n = 3), and methyl bipyrroles (MBPs; n = 1) (Fig 1). Our detection of numerous PCB, PBDE, and OC pesticides demonstrates this nontargeted method can reliably detect currently monitored compounds, and our detections of these compounds are consistent with other studies in San Diego Bay [19, 26, 27].

### Compound identification and relative abundance

#### Typically monitored compounds

Typically monitored contaminants are those that are routinely monitored in marine environmental surveys of biota. Within the southern California marine environment, these typically monitored compounds included various organochlorine pesticides (i.e., *p*,*p’*-DDE, mirex, dieldrin), PCBs, and PBDEs.

Our approach identified 84 typically monitored compounds that were verified via authentic mass spectrum match (from a synthetic standard run using the same instrumental method), retention times, and/or reference database mass spectrum (see Methods). These include 11 organochlorine pesticides; 67 individual PCBs total, with 31 congeners confirmed with synthetic standards (PCB 31/28, 49, 52, 56/60, 74, 95, 97, 99, 101, 105, 110, 128, 132, 138, 141, 149, 153, 156, 167, 170, 180, 183, 187, 194, 195, 201, 203, 206, 209) and 36 congeners identified by mass spectral similarity; and 9 known PBDE congeners (BDE 17/25, 28, 47, 66, 99, 100, 153, 154) confirmed by synthetic standards. Because our study aims to highlight compounds that are considered typically unmonitored or unknown, the typically monitored compounds mentioned above will not be discussed in detail (Table 1). However, we note that all confirmed PCB and PBDE congeners detected in our samples are consistent with those documented in the seabird ecotoxicological literature [28–31].

**Table 1:** Compound Detection Frequency Per Egg, excluding PCBs or PBDEs.

Class	Compound	T/U*	ID^#^	A	B	C	D
**OC Pesticides**	trans-Chlordane	T	1	x	x	x	x
	cis-Chlordane	T	1	x		x	
	trans-Nonachlor	T	1	x	x	x	x
	cis-Nonachlor	T	1	x	x	x	x
	Chlordane Related 1	U	4	x	x	x	x
	Chlordane Related 2	U	5	x	x	x	x
	p,p'-DDE	T	1	x	x	x	x
	Ethane, 1,1-bis(p-chlorophenyl)- (DDeT)	U	3	x	x		
	p,p’-DDMU	T	1	x	x	x	x
	DDT-related 1	U	1	x	x	x	
	DDT-related 2	U	3	x	x		
	DDT-related 3	U	3	x	x	x	x
	DDT-related 4 (DDE isomer)	U	2	x	x	x	
	TCPM Tris(4-chlorophenyl)methane	U	1	x	x	x	x
	TCPMOH Tris(4-chlorophenyl)methanol	U	1	x	x	x	x
	Dieldrin	T	1	x	x	x	x
	Hexachlorobenzene	T	1	x	x	x	x
	β-Hexachlorocyclohexane	T	1	x	x	x	x
	Heptachlor epoxide	T	1	x	x		x
	Mirex	T	1	x	x	x	x
**Phenols—Anthropogenic**	4-Chlorocatechol	U	1	x	x	x	
	4-Chlorocatechol isomer	U	2	x	x	x	
	p-Chloroanisole	U	3	x		x	x
	Parachlorophenol	U	3	x	x	x	
	Triclosan	U	1	x	x	x	x
**Phenol—Natural**	4-Bromophenol	U	1	x	x	x	x
**Phenol—Mixed**	2,4,6-Tribromoanisole	U	1	x		x	
**Pyrethroids**	Cypermethrin	U	3	x			
	Permethrine 1	U	1	x			x
	Permethrine 2	U	1	x			x
**MBPs**	Heptachloro-1'-methyl-1,2'-bipyrrole	U	1	x	x	x	x
**Other Halogens**	2-Bromo-1,3-diphenyl-1,3-propanedione	U	1	x	x	x	x
	p,p'-Dichlorodiphenyl sulfone	U	1	x		x	x
	Methylmercuric iodide	U	1	x	x	x	
**Unknowns**	Unknown 1	U	7	x	x	x	x
	Unknown 2A	U	7	x	x	x	x
	Unknown 2B	U	7	x	x	x	x
	Unknown 3	U	7	x	x	x	x

*T indicates typically monitored compounds and U indicates typically unmonitored compounds;

^#^Identification confidence where (see the Methods section for full descriptions) 1 is [authentic MS RT], 2 is [authentic MS], 3 is [reference database MS], 4 is [literature MS], 5 is [manual-congener group], 6 is [manual], and 7 is [unknown]. Blanks indicate non-detect. PCBs and PBDE detections are included in S2 Table.

#### Typically unmonitored compounds

For our study, typically unmonitored compounds are those that are not widely documented in this region and matrix, contain halogens, and have relative peak intensities comparable to the typically monitored contaminants. Our method identified 27 typically unmonitored HOCs (Table 1). Confidence in the identification varied because authentic standards were not available for all compounds: 14 compounds were classified as [authentic MS RT], five compounds [reference database MS], one compound [literature MS], three compounds [manual congener group], and four compounds [unknowns]. Further information regarding these typically unmonitored compounds, their sources, and their presence in the literature is discussed in the following paragraphs, and in S2 File. These compounds are largely undocumented in California seabirds, and/or are not currently documented as bioaccumulating in tissue. Their presence in seabird eggs is indicative of bioaccumulation, maternal contaminant transfer, and potentially biomagnification.

**Fig 2:**
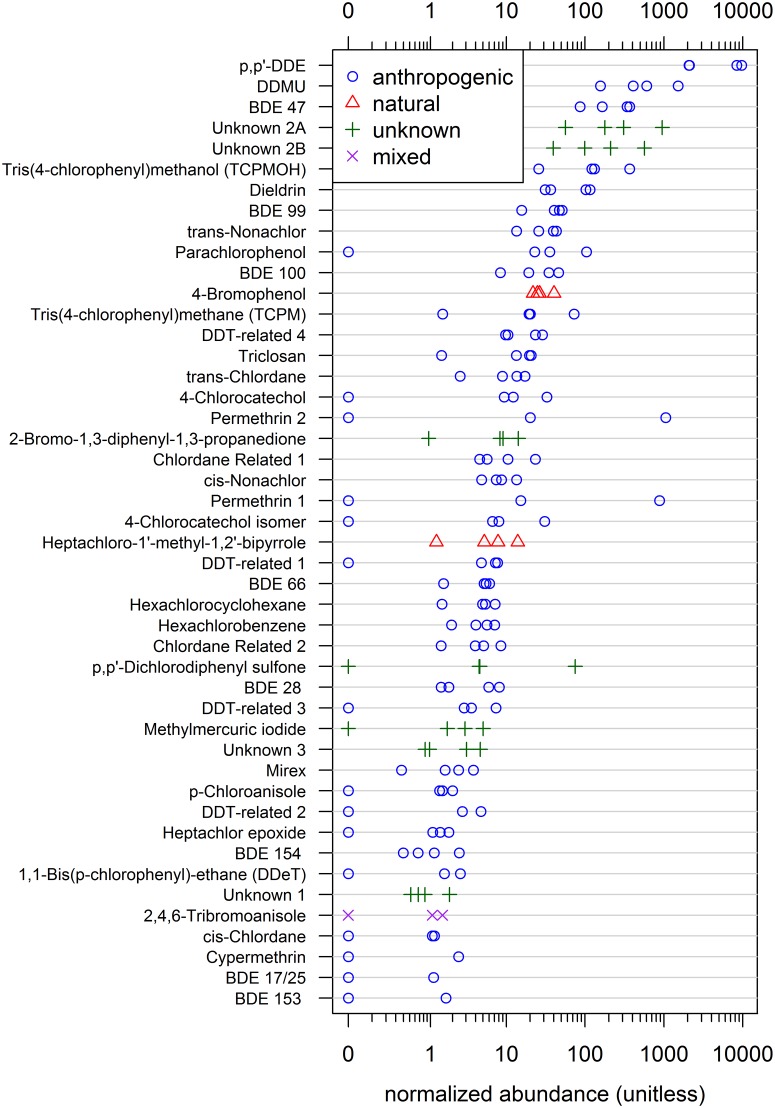
Relative Abundance of Individual Compounds, excluding PCBs. Each point represents one egg sample (n = 4), including non-detects. Non-detects are shown with a value of zero (multiple non-detects overlap). PCBs are included in S1 Fig.

Nine OC pesticides were detected. Tris(4-chlorophenyl)methane (TCPM) and tris(4-chlorophenyl)methanol (TCPMOH) were in all egg samples, which represents one of only a handful of documented reports of these compounds’ occurrence in bird tissue [30, 32]. These were among the most abundant compounds, comparable to DDMU, dieldrin, and various PCB and PBDE congeners (Fig 2 and S1 Fig). The primary source of TCPM and TCPMOH in the environment is unknown. However, possible origins of TCPM include its use in production of synthetic polymers and lightfast dyes, and its presence as a trace byproduct of technical organochlorines such as DDT and Dicofol [33, 34]. In a study by Holmstrand et al., results from the compound-specific Cl-isotope analysis (CSIA-Cl) of TCPM suggested it is of anthropogenic origin, additionally, the δ^37^Cl value for TCPM closely resembled that of technical DDT [31]. These finding support the hypothesis that TCPM is a byproduct of DDT synthesis. TCPMOH was first detected in the marine environment in 1989 [35] followed by TCPM in 1992 [33, 36]. They have since been documented in Atlantic common dolphin [10], beluga [37], California sea lions [38], and various seabird eggs [32]. Given the structural similarities of TCPM and TCPMOH to DDT, a well-documented endocrine disrupting chemical, these compounds may also exhibit adverse effects in wildlife. Two chlordane-related compounds were detected in all eggs; these compounds were previously documented in Atlantic common dolphin blubber in 2012 [10]. Chlordane Related 1 is likely to be U82, 1-exo,2-endo,3-exo,4,5,6,8,8-octachloro-3a,7,7a-tetrahydro-4,7-methanoindane, based on mass spectra similarity. U82 has been previously detected in ambient air, marine biota, and human adipose tissue; this compound is known to accumulate and persist in both humans and wildlife [39]. Ethane, 1,1-bis(*p*-chlorophenyl)- is an insecticide relative of DDT; its presence in two egg samples is worthy of further investigation. Four additional compounds were identified as DDT-related (pages, 3, 4, 8, and 9 in the SI mass spectral library) based on their retention times, mass spectra, and comparison to technical DDT standard. DDT-related 1 and DDT-related 2 contained ion clusters at *m/z* 139 and *m/z* 111, which correspond to chlorobenzaldehyde and chlorobenzene, respectively. These ion clusters are commonly seen in DDT-related compounds from previous studies [10, 40].

Seven phenols were detected, five of anthropogenic origin and two of natural origin. Triclosan is a controversial antibacterial phenolic compound discovered in all egg samples. Numerous studies have documented its uptake by plants [41], and more recent studies have documented its bioaccumulation in and toxicity to marine benthic invertebrates [42]; however, no current studies address toxicity to birds. Due to its perceived bioaccumulative properties and increasing linkages to endocrine disruption and decreased aquatic ecosystem health, it is currently under review by the US Food & Drug Administration. Parachlorophenol is a pesticide used to control various fungi, bacteria, algae, and viruses. It is highly toxic to aquatic organisms, but is not known to be toxic to birds (US EPA, 1995 http://www.epa.gov/oppsrrd1/REDs/factsheets/2045fact.pdf); in our samples, it was the most abundant phenol. 4-Bromophenol, detected in our four samples, is present in polychaetes and algae and thought to cause the typical sea-like taste and flavor; however, there is limited knowledge of its toxicological effects. 2,4,6-Tribromoanisole (TBA), the metabolite of 2,4,6-Tribromophenol (TBP), was detected in two egg samples. Both natural and anthropogenic sources exist for TBP and TBA. TBP has been used as a flame-retardant and is a breakdown product of polybrominated flame-retardants [43]. Currently, TBA is used as a fungicide on wood pallets and packaging materials [44]. Although TBP is also naturally present and ubiquitous in marine food webs [45], a recent study showed it affects reproduction in fish [46].

Three pyrethroids were detected. Pyrethroids are neurotoxic insecticides known to be toxic to fish, but are purportedly converted to non-toxic metabolites in birds and mammals. A recent study demonstrated pyrethroid bioaccumulation in dolphins, as well as maternal transfer of these compounds [47]. Heptachloro-1'-methyl-1,2'-bipyrrole was detected in all four samples. Methyl bipyrroles (MBPs) are a group of halogenated natural products known to bioaccumulate and exhibit physical-chemical properties similar to anthropogenic POPs; the precise source is unknown [48]. Heptachloro-1'-methyl-1,2'-bipyrrole is a natural MBP first documented by Vetter [49], and was detected in all egg samples. It has also been detected in marine mammals, Antarctic air, various seabird eggs, and human breast milk [10, 50–52].

Three additional halogenated compounds were detected in a majority of samples: methylmercuric iodide, 2-bromo-1,3-diphenyl-1,3-propanedione, and *p*,*p’*-dichlorodiphenyl sulfone. Little toxicity and source information is available for these compounds. Of particular interest was methylmercuric iodide, which was initially identified through a library match, then confirmed with an authentic standard. Methylmercury is known to adversely affect reproduction and the behavior of aquatic birds. In tissues, methylmercury may form many different complexes with non-metallic elements [53]. Methyl iodide is a naturally occurring marine compound released by seaweed and algae as a defense chemical; however, its role in methylating mercury is still unclear [54].

#### Unknown compounds

Four unknown compounds containing halogen isotope clusters were identified in the samples. These unknown compounds had similar or larger relative chromatographic peak intensities compared to currently monitored contaminants, indicating the potential for bioaccumulation. As a class, the relative abundance was similar to the major POP classes and was higher than PBDEs, which as a class was made up of 9 congeners (Fig 1). In particular, Unknown 2A and Unknown 2B (Fig 2) had high relative peak areas. More information on these unknowns, including their mass spectra, can be found in the SI mass spectral library.

#### Limitations and future work

Compound identification was challenging due the limited availability of authentic standards. Thirteen compounds were tentatively identified by similarity to a reference spectrum. The tentative identification of compounds with only one halogen and one phenyl group, chlorophenol and chloroanisole, has lower confidence (there are fewer fragment ions on which to base the identification). Future work should verify tentatively-identified compounds of interest. In addition, sample preparation affects compound detection [11]. Optimization of the sample clean-up procedure is necessary to maximize the type and number of identified compounds. Aggressive clean-up may eliminate compounds of interest. We used non-destructive GPC to remove lipids, instead of sulfuric acid digestion, which may break down contaminants of interest. However, it is possible that compounds of interest were lost during extraction, where 6N HCl was used for protein precipitation.

## Conclusions

Seabirds, especially piscivores feeding at upper trophic levels, may accumulate contaminants in higher concentrations than other marine animals, and as a result potentially serve as indicators of coastal health and of contaminants in other high-level consumers. Many targeted ecotoxicological studies in avifauna have demonstrated adverse and sub-lethal health consequences associated with known, POPs (i.e. PCBs, PBDEs, DDTs, etc.), which were present and abundant in all Black skimmer egg samples in our study. Most importantly, however, detection of toxic compounds in seabird eggs is indicative of bioaccumulation and is a direct result of maternal transfer via dietary intake. In addition to the detection of known POPs, our research identified 27 previously unreported or unknown compounds in bird eggs, including TCPM, TCPMOH, triclosan, permethrin, and four unknown HOCs. Although permethrin is not thought to bioaccumulate in higher order organisms, our findings challenge this assertion and corroborate recent research [46]. Furthermore, triclosan, which is harbored in soil, may be terrestrially absorbed [55] via substrates upon which birds nest.

Targeted contaminant studies focusing on typically monitored POPs are necessary and have provided the bulk of our ecotoxicological knowledge to date. However, without the addition of new compounds to these targeted studies, it is impossible to track novel contaminants. Our study represents the first comprehensive nontargeted contaminant study for bird eggs, and results from our study both complement and advance the findings of Hoh et al. [10], demonstrating that the nontargeted GC×GC/TOF-MS analytical method can provide a robust solution to identifying both known compounds and unexpected contaminants.

Nontargeted methods are an integral step in moving from reactive to proactive ecotoxicological studies, as they can identify potentially hazardous compounds as they are emerging in the environment and suggest new compounds to be included in future targeted contaminant surveys. Based on these findings, we advocate that future studies in wildlife ecotoxicology employ a nontargeted analytical approach to improve our knowledge of contaminant occurrence and of environmental risk.

## Supporting Information

S1 Table GC×GC/ToF-MS Analysis Conditions. (DOCX)

S2 Table Complete Compound Detection Frequency Per Egg. (DOCX)

S1 Fig Relative Abundance of Individual Compounds. (TIFF)

S1 File San Diego Bay Black Skimmer Egg Mass Spectral Library. (PDF)

S2 File Supporting Methods and additional information. (PDF)
