# Supplementary material for: Identifying Bioaccumulative Halogenated Organic Compounds Using a Nontargeted Analytical Approach: Seabirds as Sentinels
Source: PLoS One. 2015 May 28;10(5):e0127205. doi: 10.1371/journal.pone.0127205 (PMC4447384; doi:10.1371/journal.pone.0127205)
Supplement: S1 Table — (DOCX) [file pone.0127205.s001.docx]

S1 Table. GC×GC/TOF-MS Analysis Conditions.

| Inlet |  | Splitless |  |  |
| --- | --- | --- | --- | --- |
| Injection volume |  | 1 μL |  |  |
| Inlet temperature |  | 300^o^C for entire run |  |  |
| Column conditions |  | 1st Dimension |  | Rtx-5 35m × 0.25mm × 0.25μm |
|  |  | 2nd Dimension |  | Rtx-17 1m × 0.10mm × 0.1μm |
| Carrier Gas |  | Helium |  |  |
| Carrier gas flow |  | Constant flow 1 mL/min |  |  |
| 1st dimension oven program |  | Initial 60^o^C for 1 min with 6^o^C/min to 300^o^C for 3 min | | |
|  |  | 20^o^C/min to 320^o^C for 15 min |  |  |
| 2nd dimension oven program |  | Initial 80^o^C for 1 min with 6^o^C/min to 320^o^C for 3 min | | |
|  |  | 20^o^C/min to 340^o^C for 15 min |  |  |
| Modulator temperature offset relative to 1st GC oven |  | 35^o^C |  |  |
|  |  | Modulation period |  | 3.5 sec |
| Modulation timing |  | Hot pulse time |  | .9 sec |
|  |  | Cool time between stages |  | .85 sec |
| Transfer mass line temperature | | 285^o^C |  |  |
| Mass Range |  | 50 to 800 |  |  |
| ToF-MS temperature |  | Ion Source |  | 250^0^C |
| Solvent delay |  | 10 min |  |  |
| Scan rate |  | 151.51 spectra/sec |  |  |
| Detector Voltage |  | 1600 |  |  |
| Electron Energy (volts) |  | -70 |  |  |
