# Supplementary material for: Identifying Bioaccumulative Halogenated Organic Compounds Using a Nontargeted Analytical Approach: Seabirds as Sentinels
Source: PLoS One. 2015 May 28;10(5):e0127205. doi: 10.1371/journal.pone.0127205 (PMC4447384; doi:10.1371/journal.pone.0127205)
Supplement: S2 Table — (DOCX) [file pone.0127205.s002.docx]

**S2 Table*.* Complete Compound Detection Frequency Per Egg.**

| **Class** | **Compound** | **T/U^*^** | **ID^#^** | **A** | **B** | **C** | **D** |
| --- | --- | --- | --- | --- | --- | --- | --- |
| **OC Pesticides** | trans-Chlordane | T | 1 | x | x | x | x |
|  | cis-Chlordane | T | 1 | x |  | x |  |
|  | trans-Nonachlor | T | 1 | x | x | x | x |
|  | cis-Nonachlor | T | 1 | x | x | x | x |
|  | Chlordane Related 1 | U | 4 | x | x | x | x |
|  | Chlordane Related 2 | U | 5 | x | x | x | x |
|  | p,p'-DDE | T | 1 | x | x | x | x |
|  | Ethane, 1,1-bis(p-chlorophenyl)- (DDeT) | U | 3 | x | x |  |  |
|  | p,p’-DDMU | T | 1 | x | x | x | x |
|  | DDT-related 1 | U | 1 | x | x | x |  |
|  | DDT-related 2 | U | 3 | x | x |  |  |
|  | DDT-related 3 | U | 3 | x | x | x | x |
|  | DDT-related 4 (DDE isomer) | U | 2 | x | x | x |  |
|  | TCPM Tris(4-chlorophenyl)methane | U | 1 | x | x | x | x |
|  | TCPMOH Tris(4-chlorophenyl)methanol | U | 1 | x | x | x | x |
|  | Dieldrin | T | 1 | x | x | x | x |
|  | Hexachlorobenzene | T | 1 | x | x | x | x |
|  | β-Hexachlorocyclohexane | T | 1 | x | x | x | x |
|  | Heptachlor epoxide | T | 1 | x | x |  | x |
|  | Mirex | T | 1 | x | x | x | x |
| **Phenols - Anthropogenic** | 4-Chlorocatechol | U | 1 | x | x | x |  |
|  | 4-Chlorocatechol isomer | U | 2 | x | x | x |  |
|  | p-Chloroanisole | U | 3 | x |  | x | x |
|  | Parachlorophenol | U | 3 | x | x | x |  |
|  | Triclosan | U | 1 | x | x | x | x |
| **Phenol - Natural**  **Phenol - Mixed** | 4-Bromophenol | U | 1 | x | x | x | x |
|  | 2,4,6-Tribromoanisole | U | 1 | x |  | x |  |
| **Pyrethroids** | Cypermethrin | U | 3 | x |  |  |  |
|  | Permethrine 1 | U | 1 | x |  |  | x |
|  | Permethrine 2 | U | 1 | x |  |  | x |
| **MBPs** | Heptachloro-1'-methyl-1,2'-bipyrrole | U | 1 | x | x | x | x |
| **Other Halogens** | 2-Bromo-1,3-diphenyl-1,3-propanedione | U | 1 | x | x | x | x |
|  | p,p'-Dichlorodiphenyl sulfone | U | 1 | x |  | x | x |
|  | Methylmercuric iodide | U | 1 | x | x | x |  |
| **Unknowns** | Unknown 1 | U | 7 | x | x | x | x |
|  | Unknown 2A | U | 7 | x | x | x | x |
|  | Unknown 2B | U | 7 | x | x | x | x |
|  | Unknown 3 | U | 7 | x | x | x | x |
| **PBDEs** | BDE 47 | T | 1 | x | x | x | x |
|  | BDE 99 | T | 1 | x | x | x | x |
|  | BDE 100 | T | 1 | x | x | x | x |
|  | BDE 66 | T | 1 | x | x | x | x |
|  | BDE 28 | T | 1 | x | x | x | x |
|  | BDE 153 | T | 1 | x |  |  |  |
|  | BDE 17/25 | T | 1 | x |  |  |  |
|  | BDE 154 | T | 1 | x | x | x | x |
| **Polychlorinated Biphenyls** | PCB 31/28 | T | 1 | x | x | x | x |
|  | PCB 49 | T | 1 | x | x | x | x |
|  | PCB 52 | T | 1 | x | x | x | x |
|  | PCB 56/60 | T | 1 | x | x | x | x |
|  | PCB 74 | T | 1 | x | x | x | x |
|  | PCB 95 | T | 1 | x | x | x | x |
|  | PCB 97 | T | 1 | x | x | x | x |
|  | PCB 99 | T | 1 | x | x | x | x |
|  | PCB 101 | T | 1 | x | x | x | x |
|  | PCB 105 | T | 1 | x | x | x | x |
|  | PCB 110 | T | 1 | x | x | x | x |
|  | PCB 128 | T | 1 | x | x | x | x |
|  | PCB 132 | T | 1 | x | x | x |  |
|  | PCB 138 | T | 1 | x | x | x | x |
|  | PCB 141 | T | 1 | x | x | x |  |
|  | PCB 149 | T | 1 | x | x | x | x |
|  | PCB 153 | T | 1 | x | x | x | x |
|  | PCB 156 | T | 1 | x | x | x | x |
|  | PCB 167 | T | 1 | x | x | x | x |
|  | PCB 170 | T | 1 | x | x | x | x |
|  | PCB 180 | T | 1 | x | x | x | x |
|  | PCB 183 | T | 1 | x | x | x | x |
|  | PCB 187 | T | 1 | x | x | x | x |
|  | PCB 194 | T | 1 | x | x | x | x |
|  | PCB 195 | T | 1 | x |  |  |  |
|  | PCB 201 | T | 1 | x | x | x | x |
|  | PCB 203 | T | 1 | x | x | x | x |
|  | PCB 206 | T | 1 | x | x |  |  |
|  | PCB 209 | T | 1 | x | x |  | x |
|  | PCB 4Cl-1 | T | 2 | x | x | x |  |
|  | PCB 4Cl-2 | T | 2 | x | x | x |  |
|  | PCB 4Cl-3 | T | 2 | x | x | x |  |
|  | PCB 4Cl-4 | T | 2 | x | x | x | x |
|  | PCB 4Cl-5 | T | 2 | x |  |  |  |
|  | PCB 4Cl-6 | T | 2 | x |  |  |  |
|  | PCB 4Cl-7 | T | 2 | x | x | x | x |
|  | PCB 5Cl-1 | T | 2 | x | x | x |  |
|  | PCB 5Cl-2 | T | 2 | x | x | x |  |
|  | PCB 5Cl-3 | T | 2 | x | x | x | x |
|  | PCB 5Cl-5 | T | 2 | x | x | x | x |
|  | PCB 5Cl-6 | T | 2 | x | x | x |  |
|  | PCB 5Cl-7 | T | 2 | x | x |  |  |
|  | PCB 5Cl-8 | T | 2 | x | x | x | x |
|  | PCB 5Cl-9 | T | 2 | x | x | x | x |
|  | PCB 5Cl-10 | T | 2 | x | x | x | x |
|  | PCB 5Cl-11 | T | 2 | x |  |  |  |
|  | PCB 5Cl-12 | T | 2 | x | x |  |  |
|  | PCB 6Cl-1 | T | 2 | x | x |  |  |
|  | PCB 6Cl-2 | T | 2 | x | x | x | x |
|  | PCB 6Cl-3 | T | 2 | x | x | x | x |
|  | PCB 6Cl-4 | T | 2 | x | x | x |  |
|  | PCB 6Cl-5 | T | 2 | x | x | x |  |
|  | PCB 6Cl-6 | T | 2 | x | x | x | x |
|  | PCB 6Cl-7 | T | 2 | x | x | x | x |
|  | PCB 7Cl-1 | T | 2 | x | x | x |  |
|  | PCB 7Cl-2 | T | 2 | x | x | x | x |
|  | PCB 7Cl-3 | T | 2 | x | x | x | x |
|  | PCB 7Cl-4 | T | 2 | x | x | x | x |
|  | PCB 7Cl-5 | T | 2 | x | x | x | x |
|  | PCB 7Cl-6 | T | 2 | x | x | x | x |
|  | PCB 7Cl-7 | T | 2 | x | x |  |  |
|  | PCB 8Cl-1 | T | 2 | x | x |  |  |
|  | PCB 8Cl-2 | T | 2 | x | x |  |  |
|  | PCB 9Cl-1 | T | 2 | x |  |  |  |
|  | PCB 9Cl-2 | T | 2 | x |  |  |  |

^*^T indicates typically monitored compounds and U indicates typically unmonitored compounds; ^#^Identification confidence where (see the Methods section for full descriptions) 1 is [authentic MS RT], 2 is [authentic MS], 3 is [reference database MS], 4 is [literature MS], 5 is [manual-congener group], 6 is [manual], and 7 is [unknown]. Blanks indicate non-detect.
