# Supplementary figures and images for: Identifying Bioaccumulative Halogenated Organic Compounds Using a Nontargeted Analytical Approach: Seabirds as Sentinels
Source: PLoS One. 2015 May 28;10(5):e0127205. doi: 10.1371/journal.pone.0127205 (PMC4447384; doi:10.1371/journal.pone.0127205)

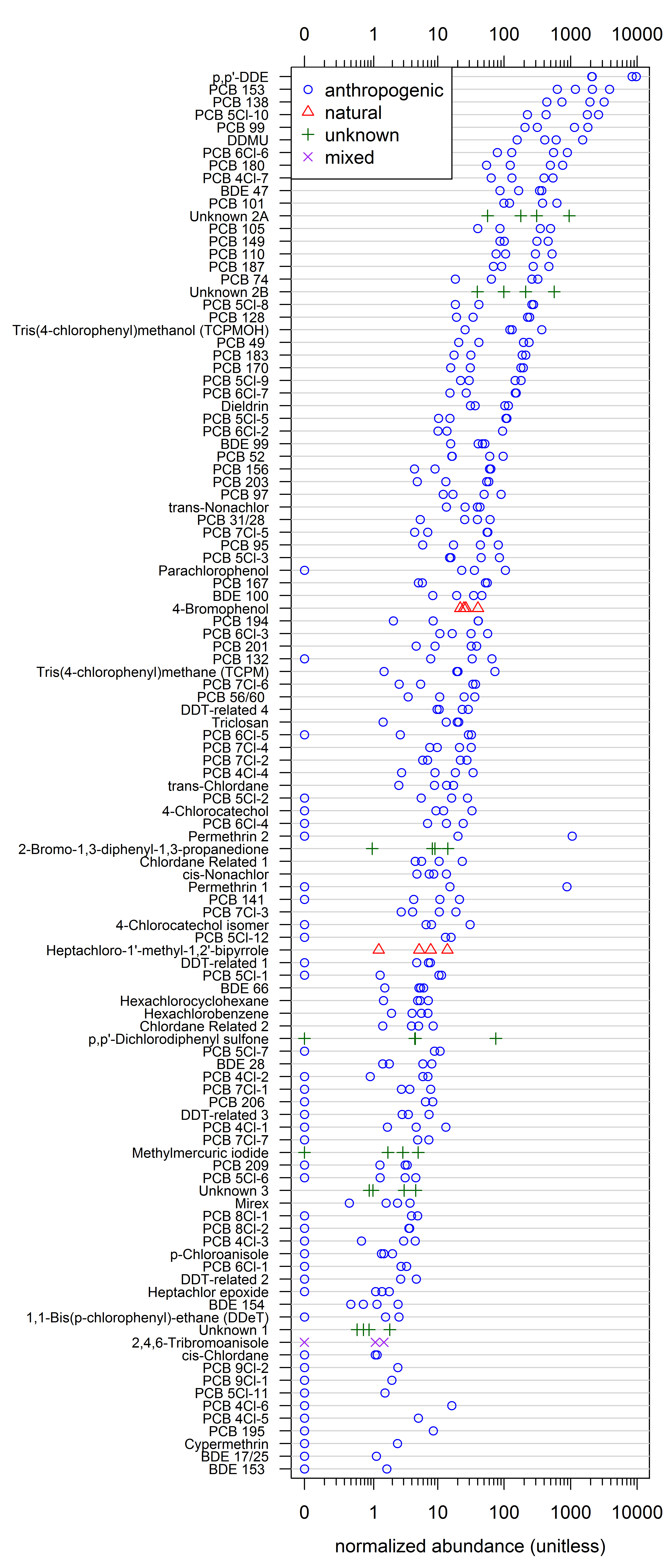

Supplement: S1 Fig — (TIFF) [file pone.0127205.s003.tiff]
