# Supplementary material for: Identifying Bioaccumulative Halogenated Organic Compounds Using a Nontargeted Analytical Approach: Seabirds as Sentinels
Source: PLoS One. 2015 May 28;10(5):e0127205. doi: 10.1371/journal.pone.0127205 (PMC4447384; doi:10.1371/journal.pone.0127205)
Supplement: S1 File — (PDF) [file pone.0127205.s004.pdf]

San Diego Bay Black Skimmer Eggs

Identifying Bioaccumulative Halogenated Organic Compounds Using a Nontargeted Analytical Approach: Seabirds as Sentinels.

Authors: Christopher Millow, Susan A. Mackintosh, Rebecca L. Lewison, Nathan G. Dodder, and Eunha Hoh

Web Reference: http://OrgMassSpec.github.io

Prepared: 2015−03−02 18:21:15
SpecLibBlackSkimmerEgg2014 version 0.1−2
OrgMassSpecR version 0.4−4
png version 0.1−7
R version 3.1.0 (2014−04−10)

Name: Heptachloro−1'−methyl−1,2'−bipyrrole

Class: MBP

Matrix: SD Bay Black Skimmer Egg
In Samples: All
Group: Not Typically Monitored
Comment: Also referred to as Q1.

Instrument: GCxGC−TOF, EI, 70 eV
1D RT, 2D RT (s): 1988.71, 1.561
Quantitative Ion m/z: 316

Elemental Formula: C9H3Cl7N2
Source: Natural
Identification: Authentic MS RT

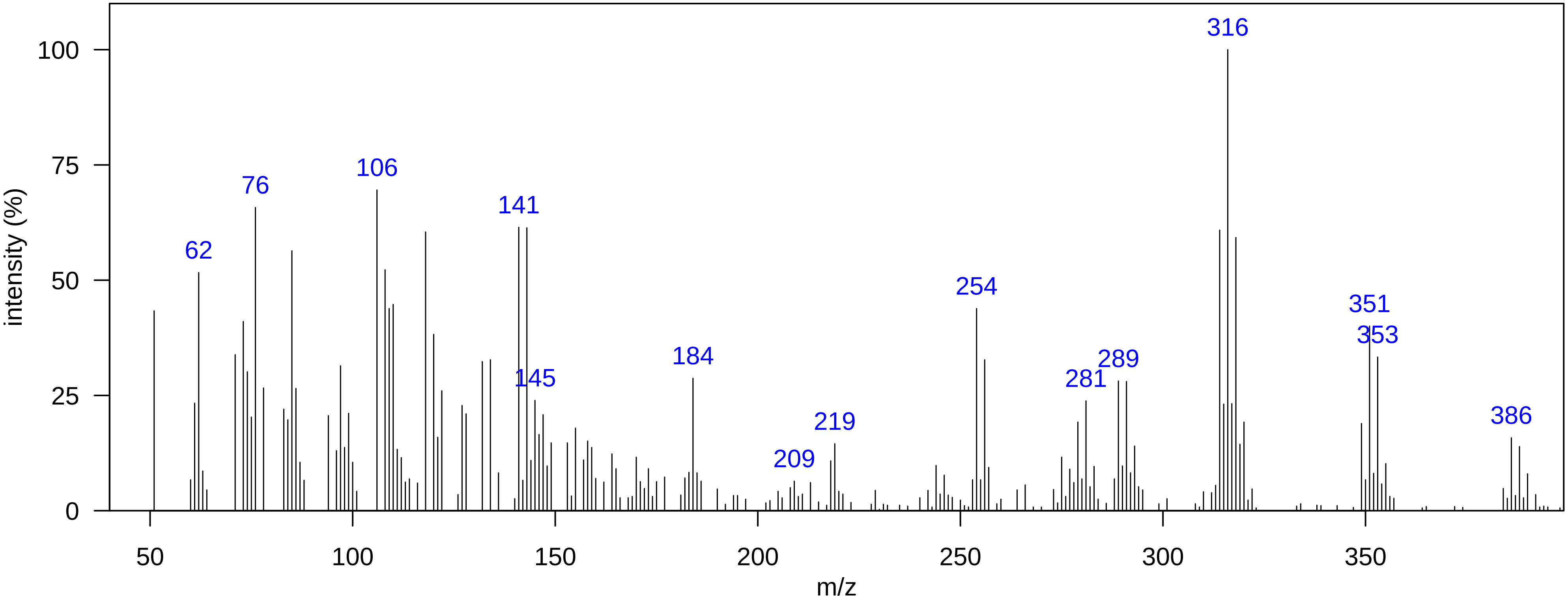

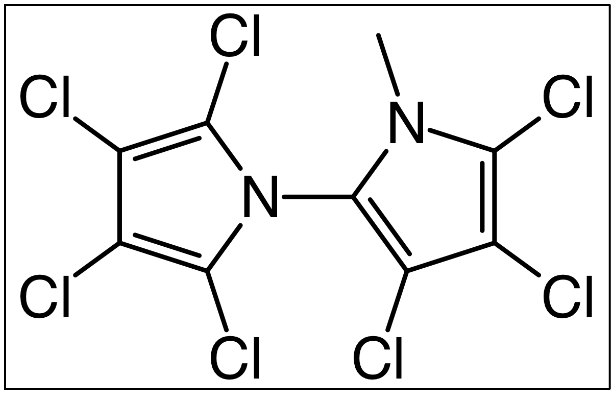

m/z [Fragment]
314 [M−Cl2]+
349 [M−Cl]+
384 M+

Name: DDT−related 1

Class: OC pesticide

Matrix: SD Bay Black Skimmer Egg
In Samples: A, B, C
Group: Not Typically Monitored
Comment: NIST I.D. is 2−Chlorobenzaldehyde. Detected in DDT tech. mix.

Instrument: GCxGC−TOF, EI, 70 eV
1D RT, 2D RT (s): 830.868, 1.32
Quantitative Ion m/z: 139

Elemental Formula: C7H5ClO
Source: Anthropogenic
Identification: Authentic MS RT

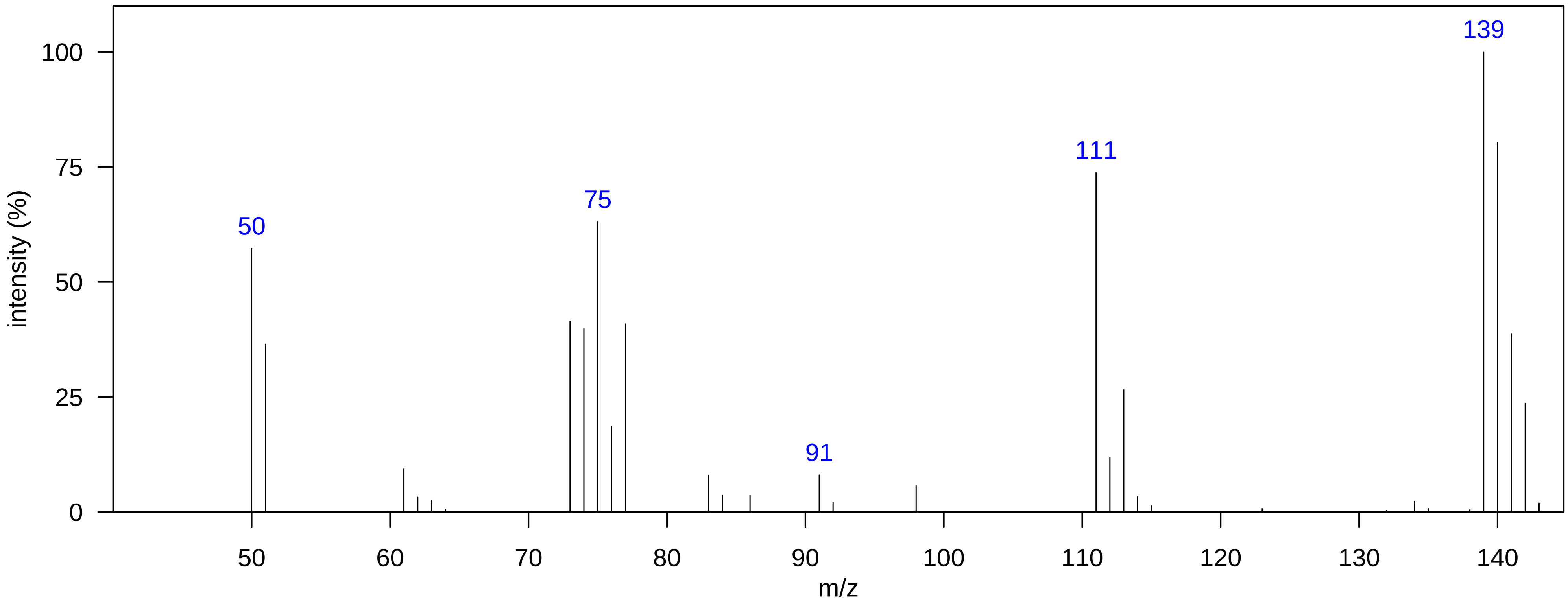

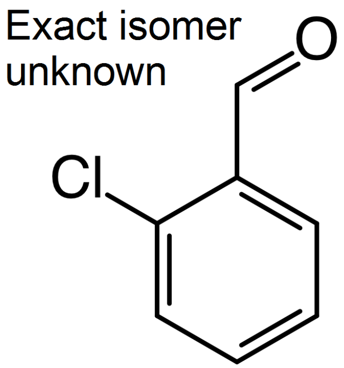

m/z [Fragment]
76 [M−CHClO]+
112 [M−CO]+
140 M+

Name: DDT−related 2

Class: OC pesticide

Matrix: SD Bay Black Skimmer Egg
In Samples: A, B
Group: Not Typically Monitored
Comment: NIST I.D. is m−Chloropropiophenone.

Instrument: GCxGC−TOF, EI, 70 eV
1D RT, 2D RT (s): 1114.21, 1.327
Quantitative Ion m/z: 139

Elemental Formula: C9H4ClO
Source: Anthropogenic
Identification: Reference Database MS

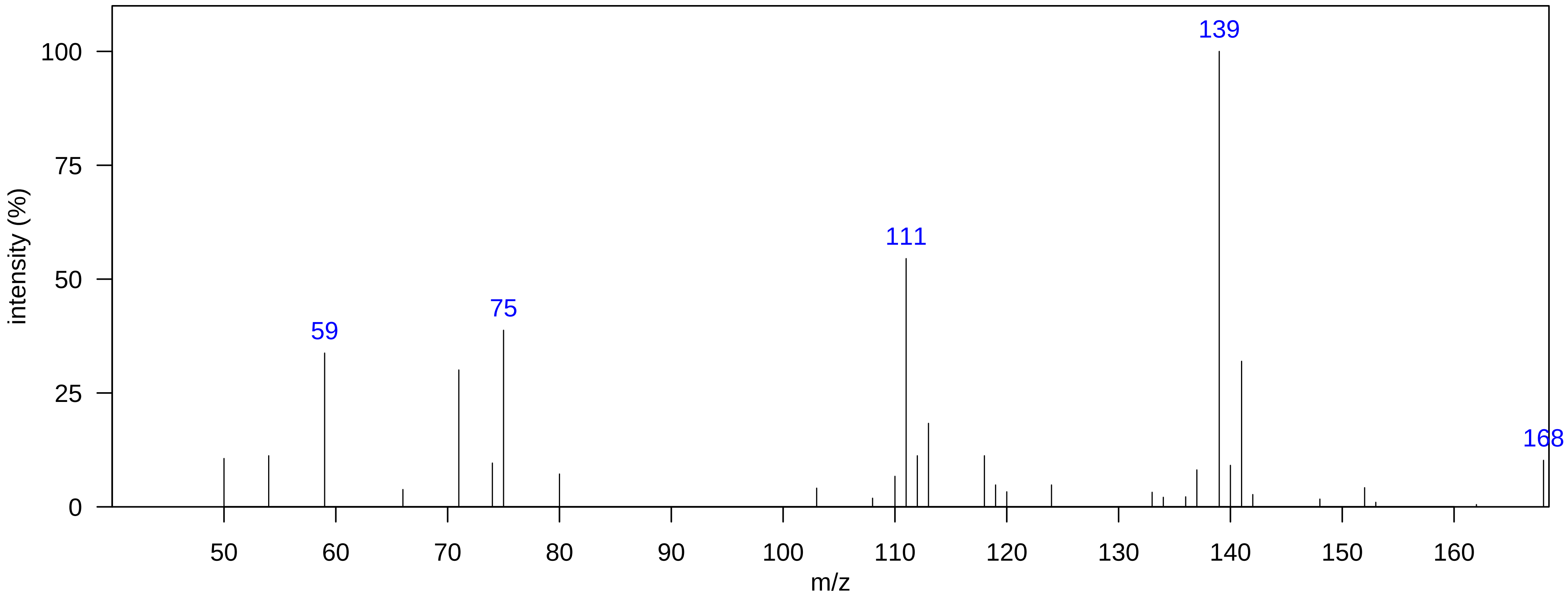

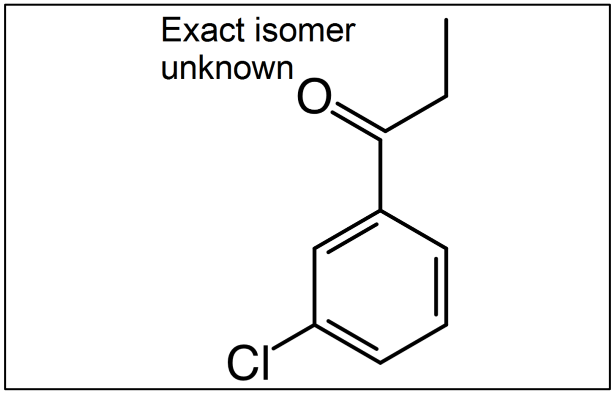

m/z [Fragment]
112 [M−C3H4O]+
140 [M−C2H4]+
168 M+

Name: 1,1−Bis(p−chlorophenyl)−ethane (DDeT)

Class: OC pesticide

Matrix: SD Bay Black Skimmer Egg
In Samples: A, B
Group: Not Typically Monitored
Comment: NIST I.D. is 1,1−Bis(p−chlorophenyl)ethane.

Instrument: GCxGC−TOF, EI, 70 eV
1D RT, 2D RT (s): 1778.83, 1.485
Quantitative Ion m/z: 235

Elemental Formula: C14H12Cl2
Source: Anthropogenic
Identification: Reference Database MS

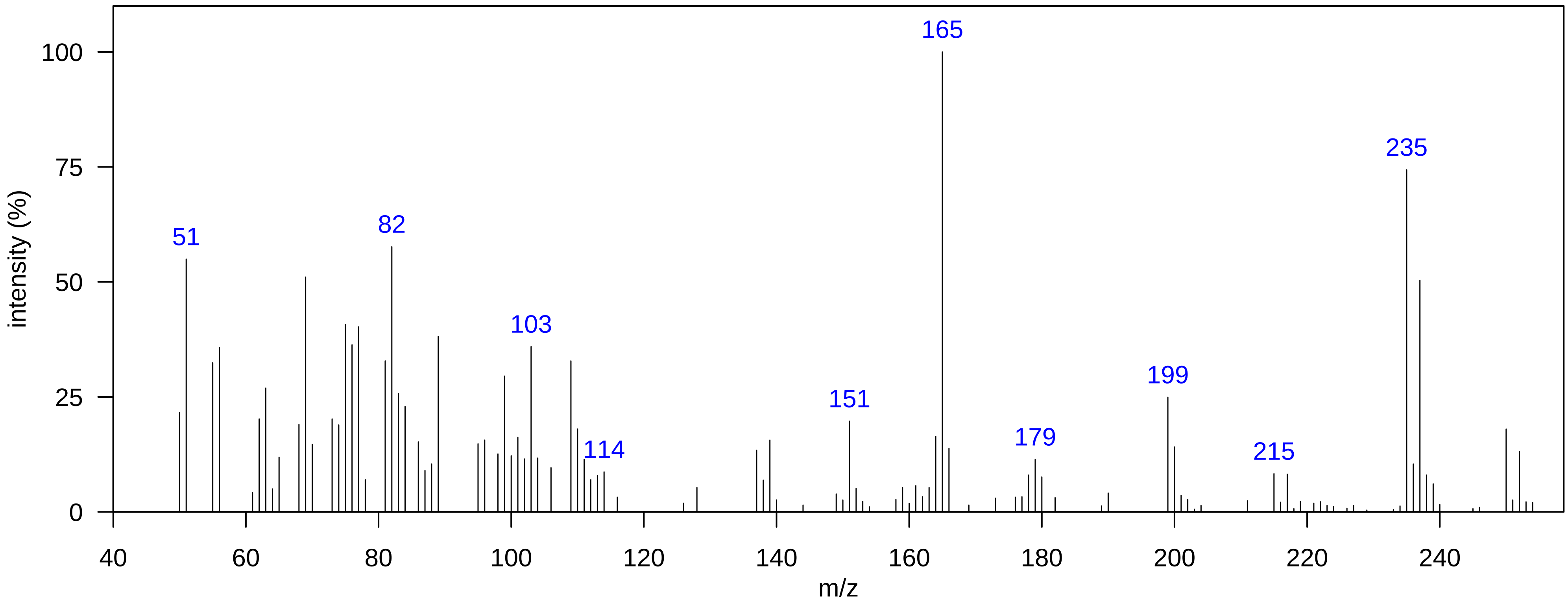

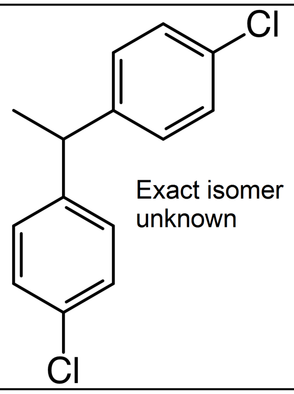

m/z [Fragment]
165 [M−CH3Cl2]+
235 [M−CH3]+
250 M+

Name: Chlordane Related 1 — Class: OC pesticide

Matrix: SD Bay Black Skimmer Egg
In Samples: All
Group: Not Typically Monitored
Instrument: GCxGC−TOF, EI, 70 eV
1D RT, 2D RT (s): 1950.23, 1.498
Quantitative Ion m/z: 238
Elemental Formula: C10H6Cl8
Source: Anthropogenic
Identification: Literature MS
Comment: M+ is not detected. Tentatively identified as U82 (Environ. Sci. Technol., 1999, 33, 1353–1358).

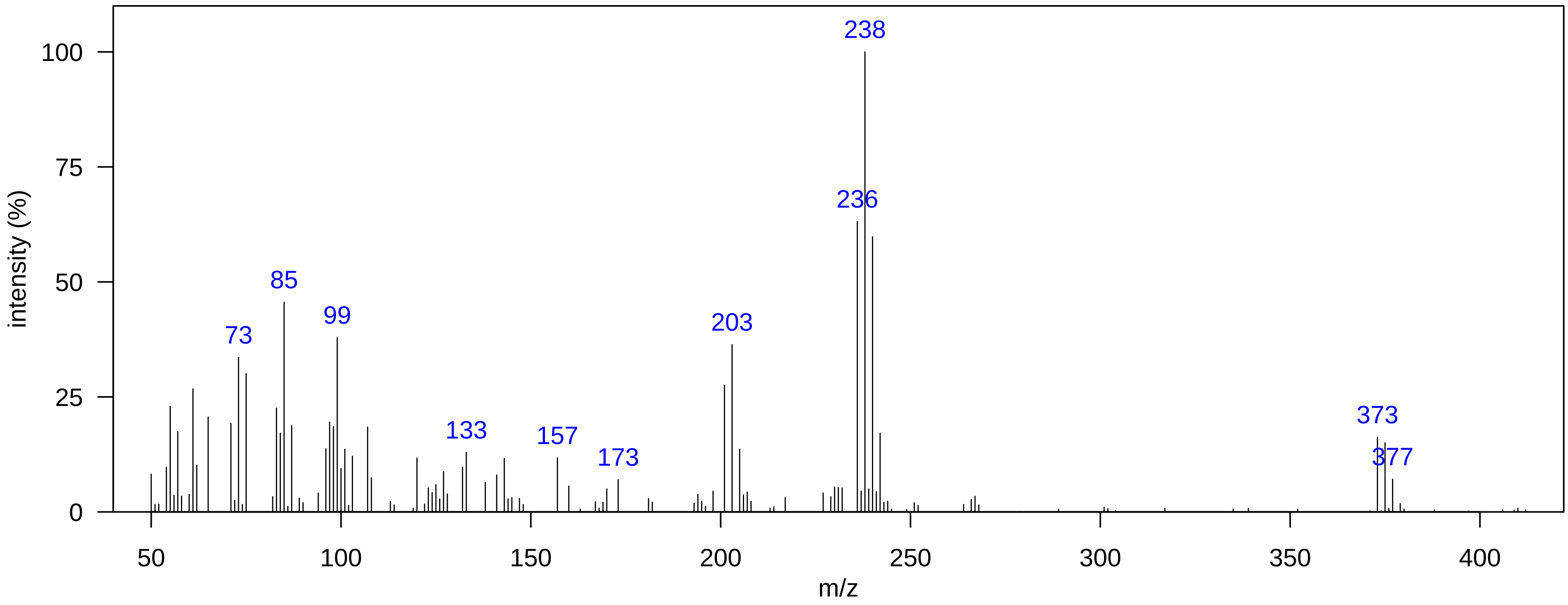

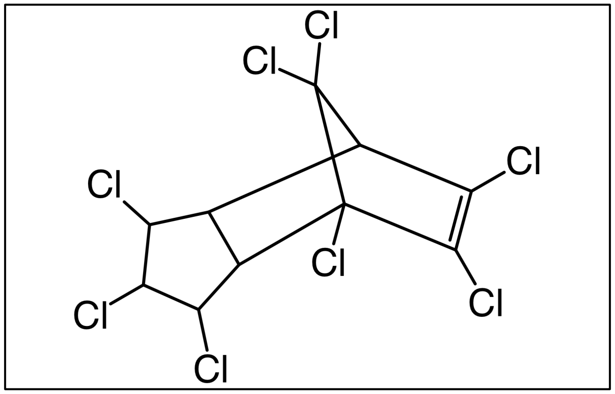

m/z [Fragment]
236 [C5HCl5]+
371 [M−Cl]+
406 M+

Name: Chlordane Related 2

Class: OC pesticide

Matrix: SD Bay Black Skimmer Egg
In Samples: All
Group: Not Typically Monitored
Comment: Ion cluster at m/z 339 suggests C10H6Cl6. M+ not detected.

Instrument: GCxGC−TOF, EI, 70 eV
1D RT, 2D RT (s): 1957.22, 1.67
Quantitative Ion m/z: 339

Elemental Formula: C10H7Cl7
Source: Anthropogenic
Identification: Manual Congener Group

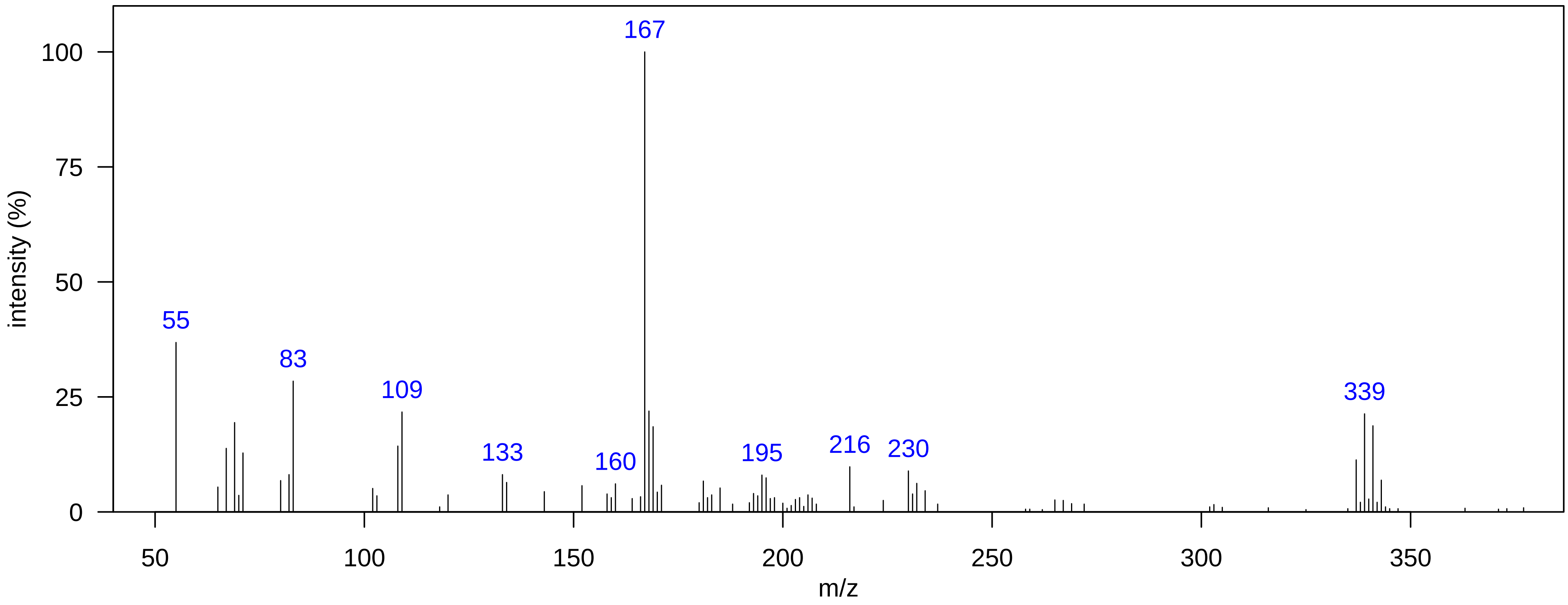

m/z [Fragment]
337 [M−Cl]+
372 M+

Name: DDT−related 3

Class: OC pesticide

Matrix: SD Bay Black Skimmer Egg
In Samples: A, B, C
Group: Not Typically Monitored
Comment: NIST I.D. is 1−Chloro−2,2−Bis(p−chlorophenyl)ethane.

Instrument: GCxGC−TOF, EI, 70 eV
1D RT, 2D RT (s): 1999.2, 1.663
Quantitative Ion m/z: 235

Elemental Formula: C14H12Cl3
Source: Anthropogenic
Identification: Reference Database MS

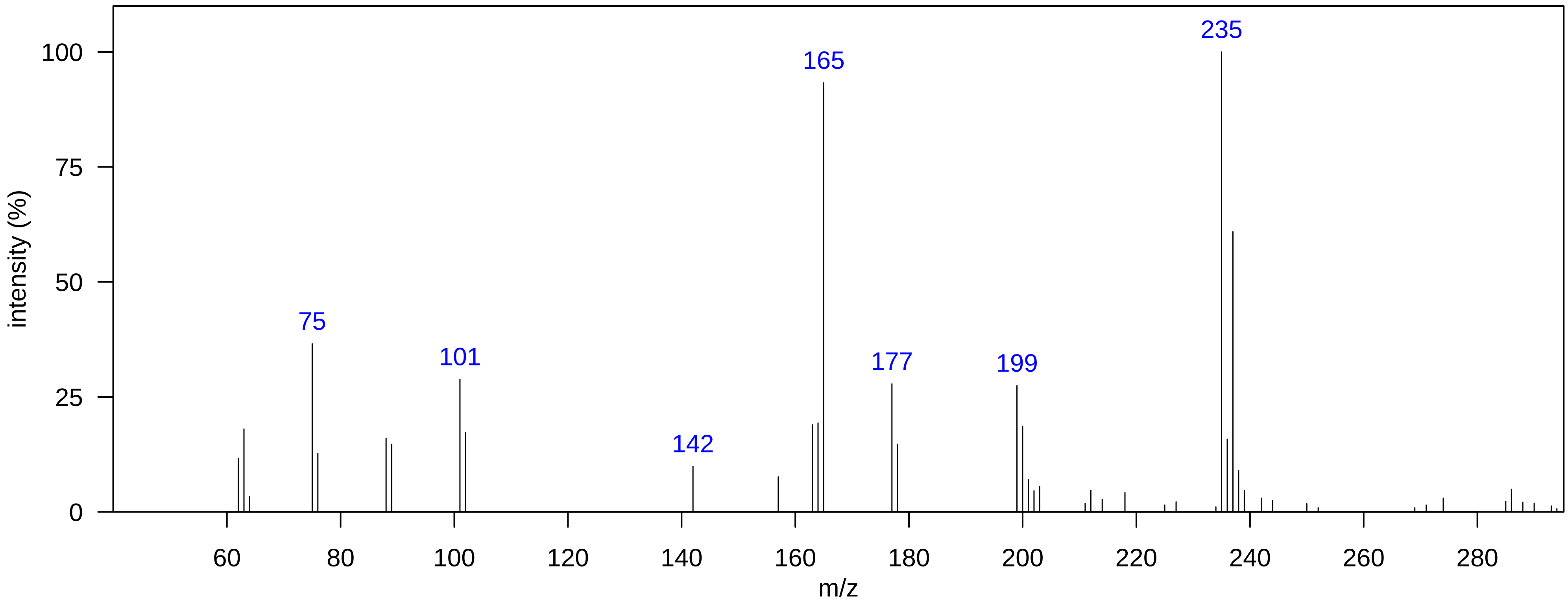

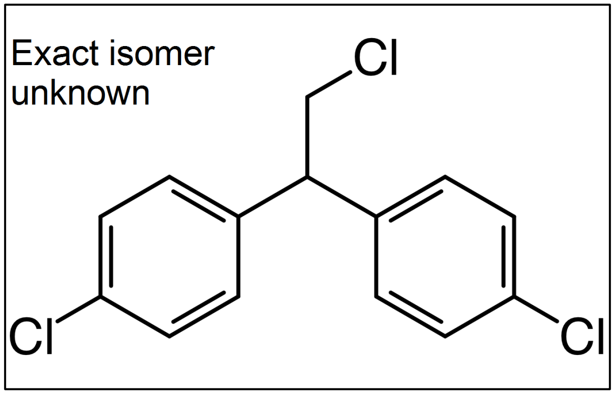

m/z [Fragment]
166 [M−CH2Cl3]+
201 [M−CH2Cl2]+
236 [M−CH2Cl]+
285 M+

Name: DDT−related 4

Class: OC pesticide

Matrix: SD Bay Black Skimmer Egg
In Samples: All
Group: Not Typically Monitored
Comment: DDE Isomer.

Instrument: GCxGC−TOF, EI, 70 eV
1D RT, 2D RT (s): 2051.67, 1.597
Quantitative Ion m/z: 318

Elemental Formula: C14H8Cl4
Source: Anthropogenic
Identification: Authentic MS

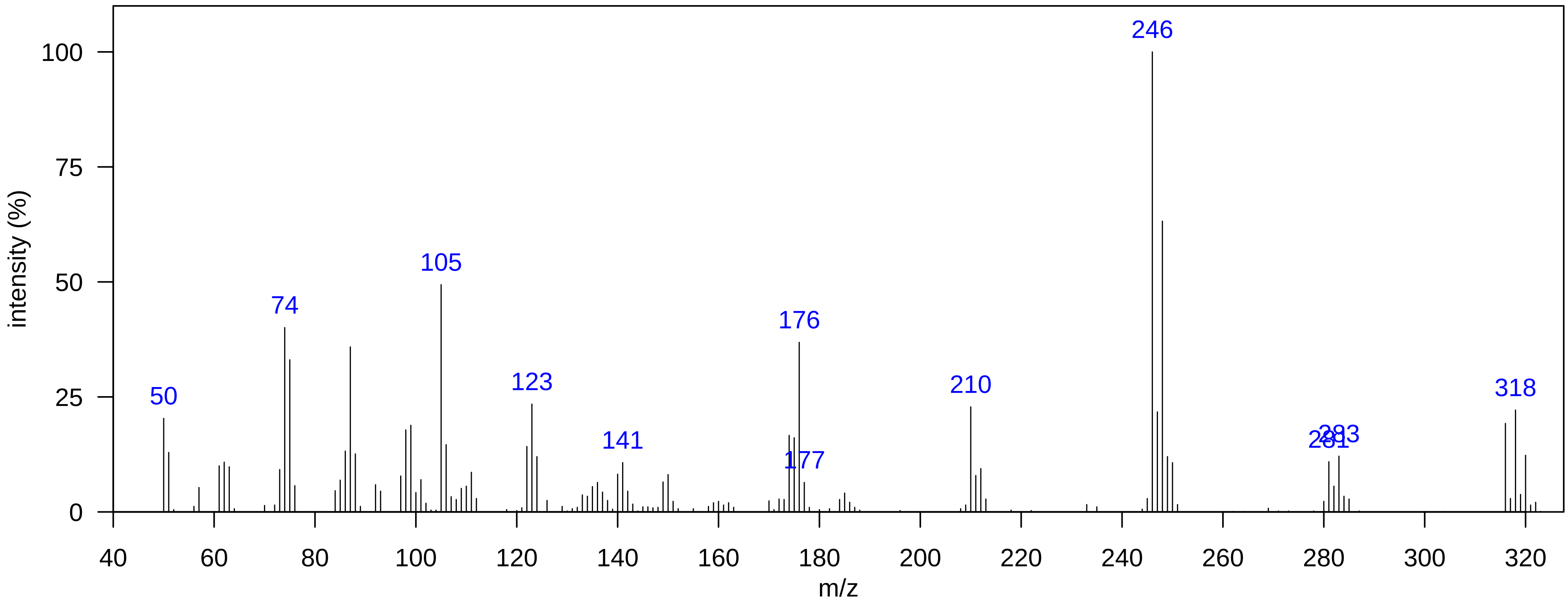

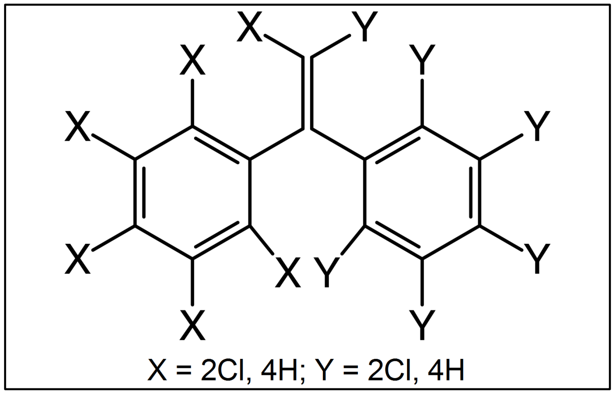

m/z [Fragment]
176 [M−Cl4]+
211 [M−Cl3]+
246 [M−Cl2]+
280 [M−Cl]+
316 M+

Name: Tris(4−chlorophenyl)methane (TCPM) Class: OC pesticide

Matrix: SD Bay Black Skimmer Egg
In Samples: All
Group: Not Typically Monitored
Comment:

Instrument: GCxGC−TOF, EI, 70 eV
1D RT, 2D RT (s): 2397.97, 1.855
Quantitative Ion m/z: 311

Elemental Formula: C19H13Cl3
Source: Anthropogenic
Identification: Authentic MS RT

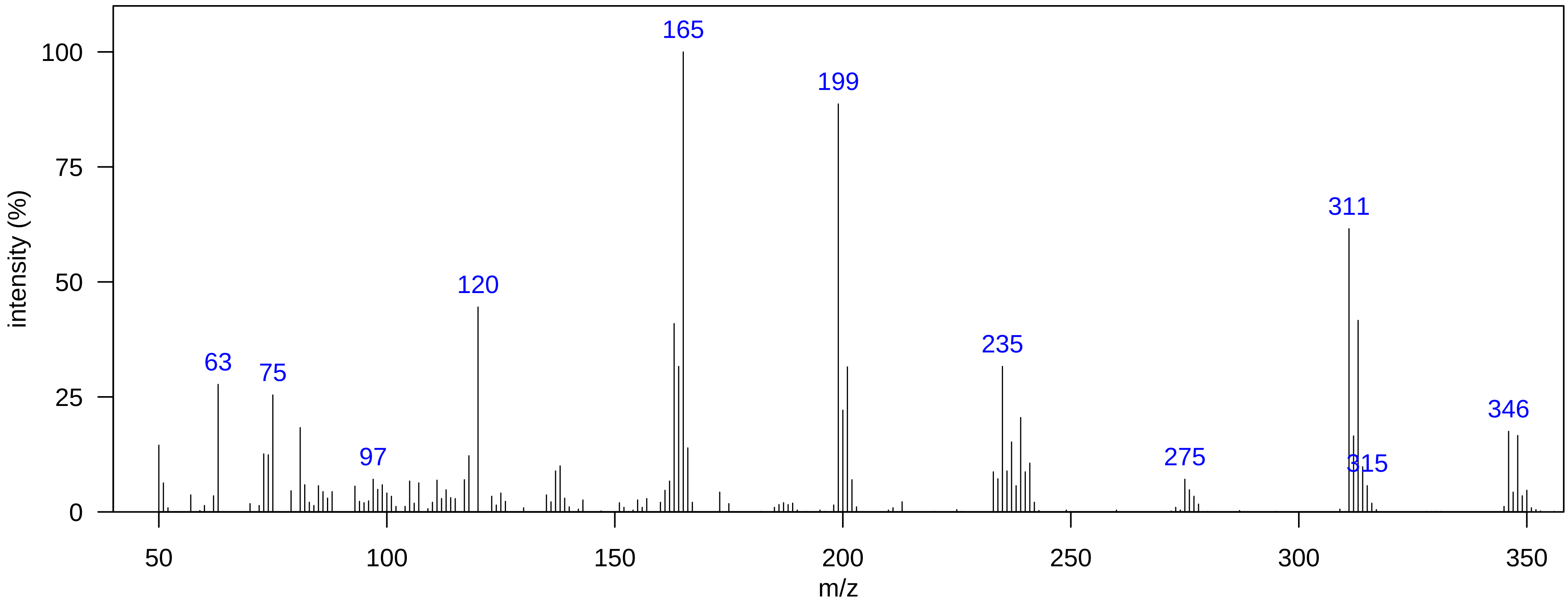

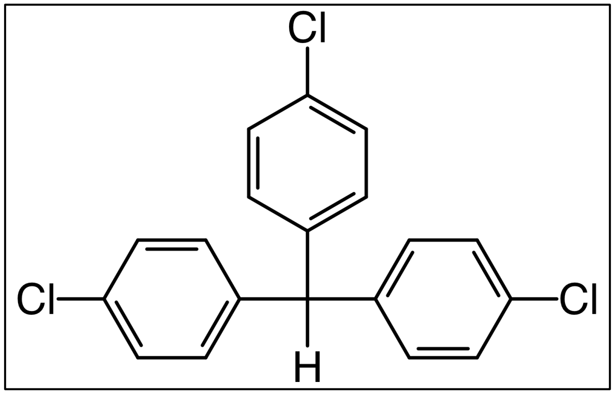

m/z [Fragment]
275 [M−HCl2]+
311 [M−Cl]+
346 M+

Name: Tris(4−chlorophenyl)methanol (TCPMOH)

Class: OC pesticide

Matrix: SD Bay Black Skimmer Egg
In Samples: All
Group: Not Typically Monitored
Comment: M+ is not detected.

Instrument: GCxGC−TOF, EI, 70 eV
1D RT, 2D RT (s): 2499.41, 2.125
Quantitative Ion m/z: 139

Elemental Formula: C19H13Cl3O
Source: Anthropogenic
Identification: Authentic MS RT

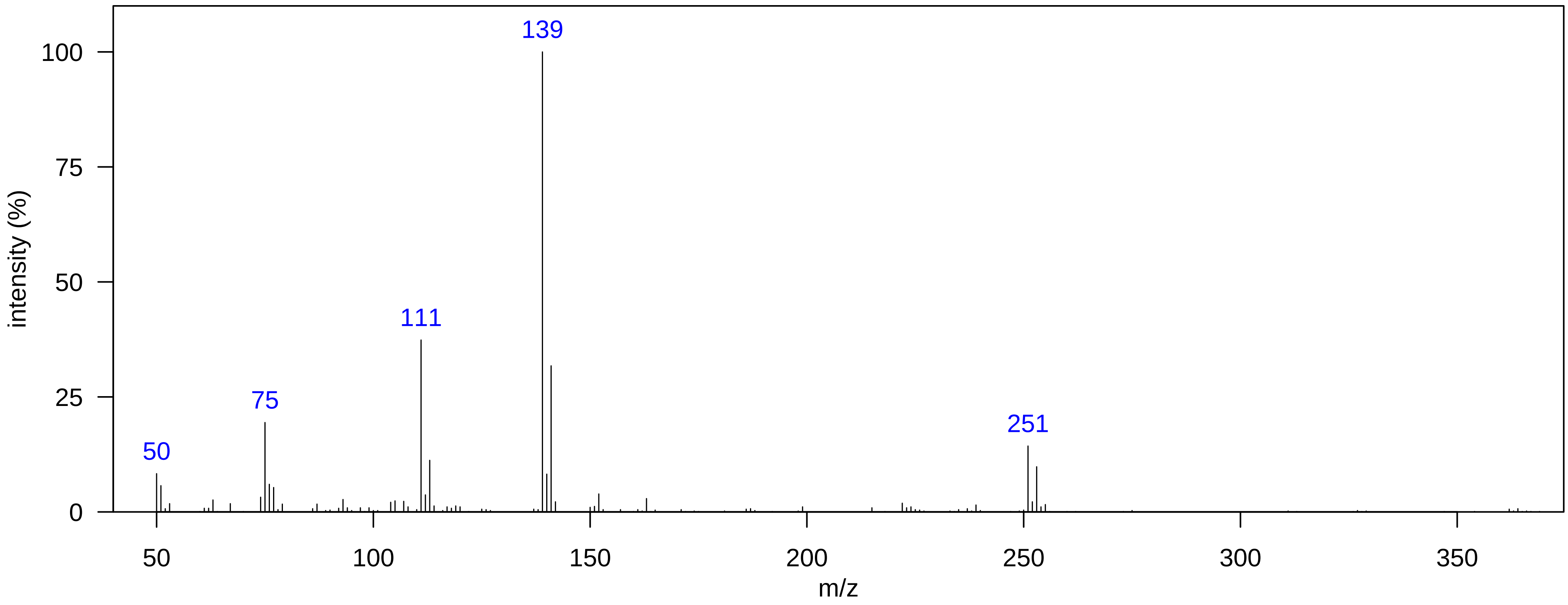

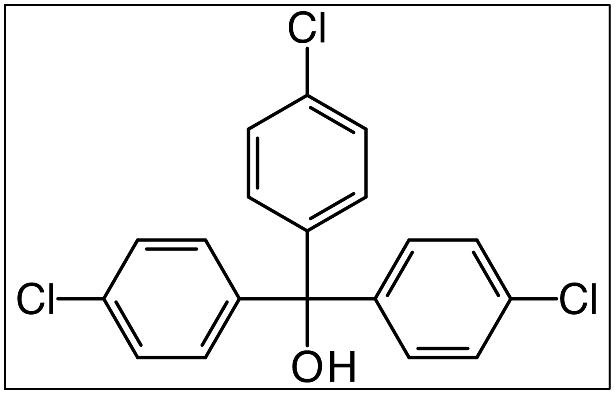

m/z [Fragment]
251 [M−C2HCl2O]+
311 [M−HClO]+
362 M+

Name: Methylmercuric iodide

Class: Other

Matrix: SD Bay Black Skimmer Egg
In Samples: A, B, C
Group: Not Typically Monitored
Comment: NIST I.D. is Methylmercuric iodide. Formula is CH3HgI.

Instrument: GCxGC−TOF, EI, 70 eV
1D RT, 2D RT (s): 753.912, 1.551
Quantitative Ion m/z: 329

Elemental Formula:
Source: Unknown
Identification: Authentic MS RT

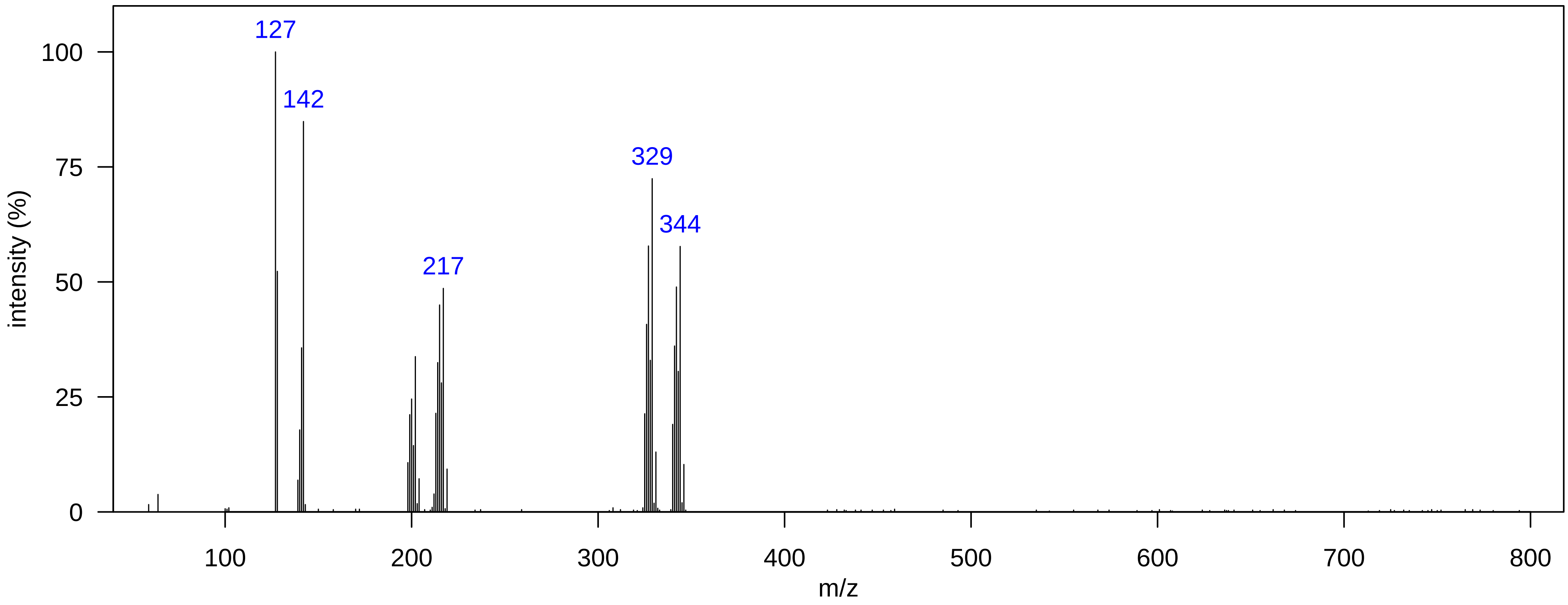

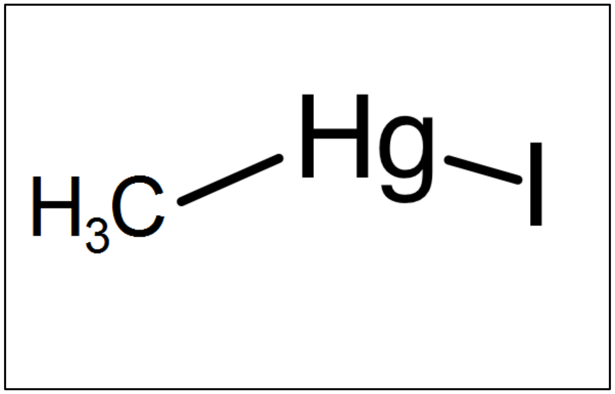

m/z [Fragment]
325 [M−CH3]+
340 M+

Name: 2−Bromo−1,3−diphenyl−1,3−propanedione

Class: Other

Matrix: SD Bay Black Skimmer Egg
In Samples: All
Group: Not Typically Monitored
Comment: M+ is not detected.

Instrument: GCxGC−TOF, EI, 70 eV
1D RT, 2D RT (s): 1932.74, 1.67
Quantitative Ion m/z: 223

Elemental Formula: C15H11BrO2
Source: Unknown
Identification: Authentic MS RT

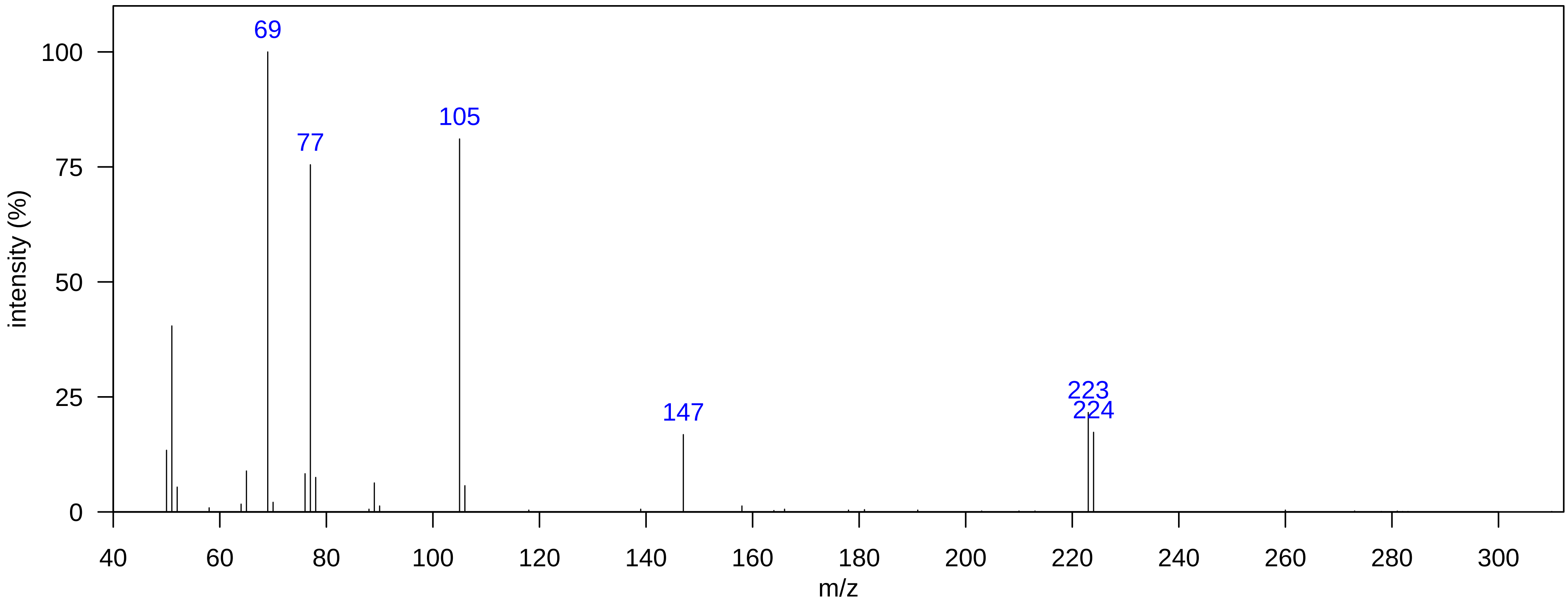

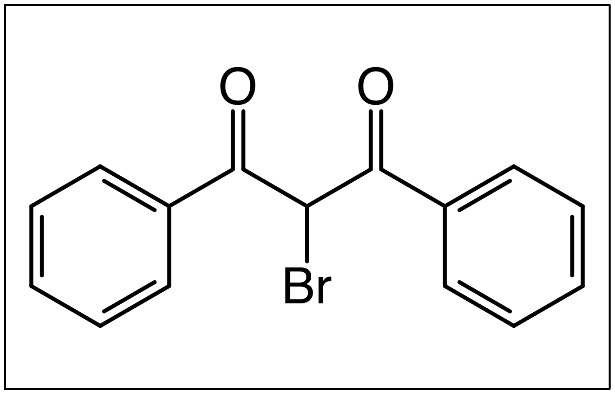

m/z [Fragment]
223 [M−Br]+
302 M+

Name: p,p'−Dichlorodiphenyl sulfone

Class: Other

Matrix: SD Bay Black Skimmer Egg
In Samples: A, C, D
Group: Not Typically Monitored
Comment:

Instrument: GCxGC−TOF, EI, 70 eV
1D RT, 2D RT (s): 2072.66, 1.855
Quantitative Ion m/z: 159

Elemental Formula: C12H8Cl2O2S
Source: Unknown
Identification: Authentic MS RT

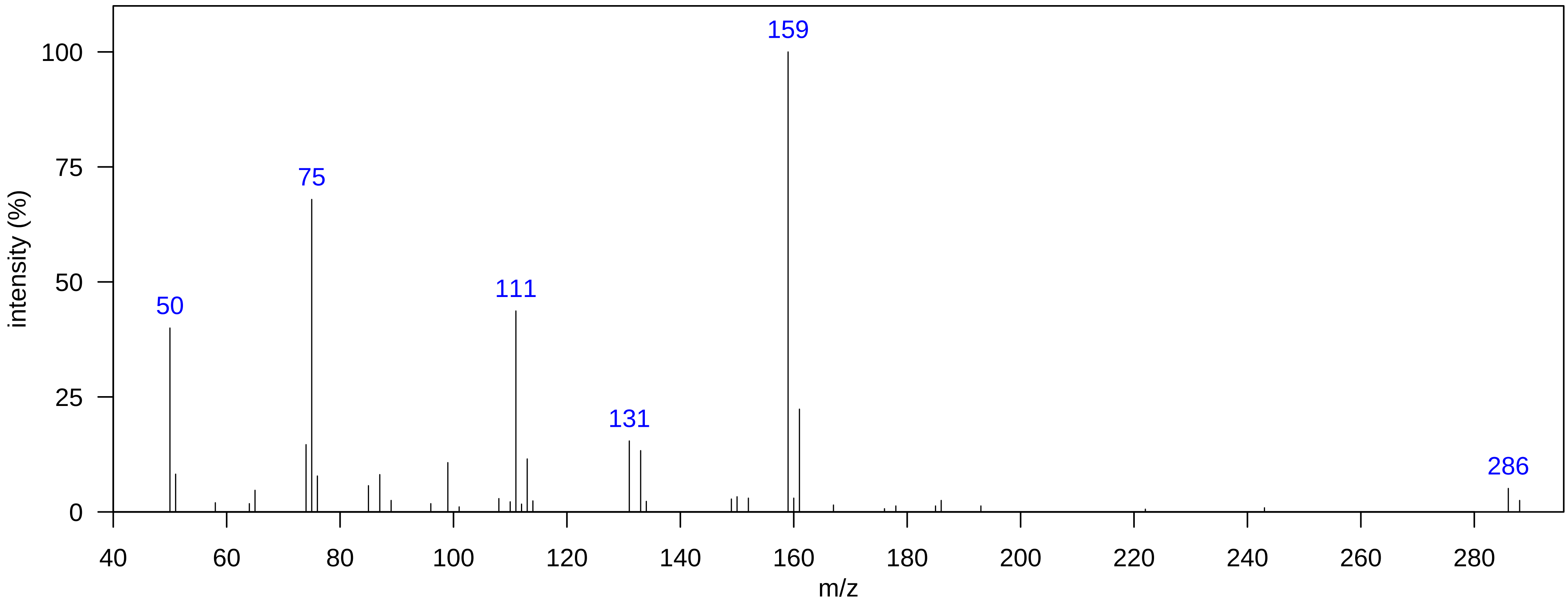

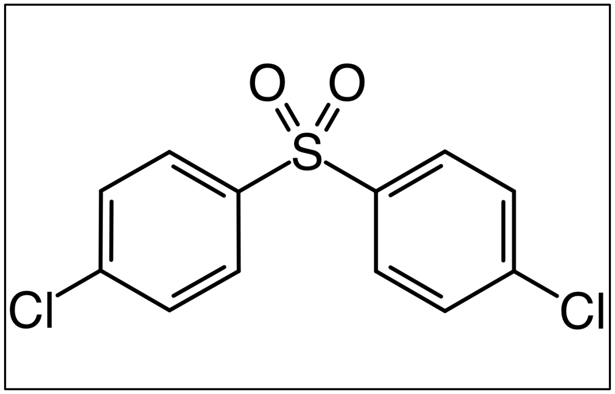

m/z [Fragment]
159 [M−C6H4ClSO]+
286 M+

Name: Parachlorophenol

Class: Phenol A

Matrix: SD Bay Black Skimmer Egg
In Samples: A, B, C
Group: Not Typically Monitored
Comment: NIST I.D. is Parachlorophenol.

Instrument: GCxGC−TOF, EI, 70 eV
1D RT, 2D RT (s): 939.306, 1.307
Quantitative Ion m/z: 128

Elemental Formula: C6H5ClO
Source: Anthropogenic
Identification: Reference Database MS

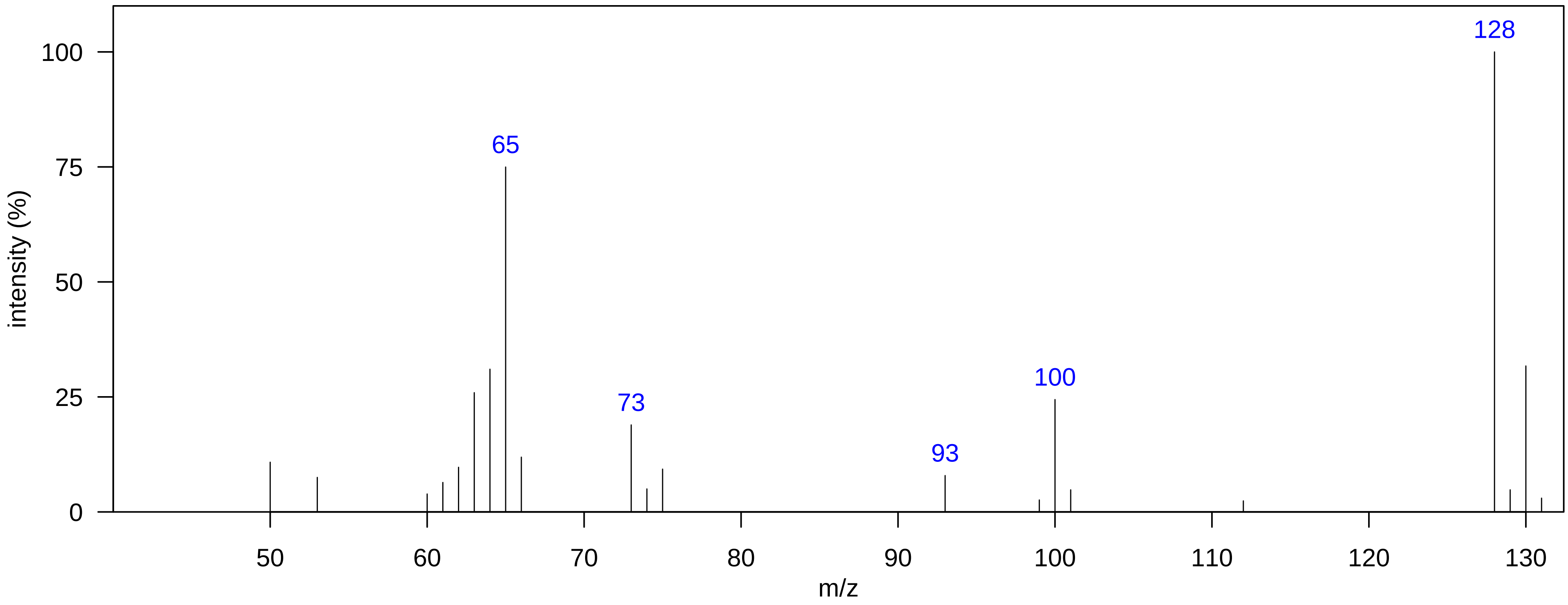

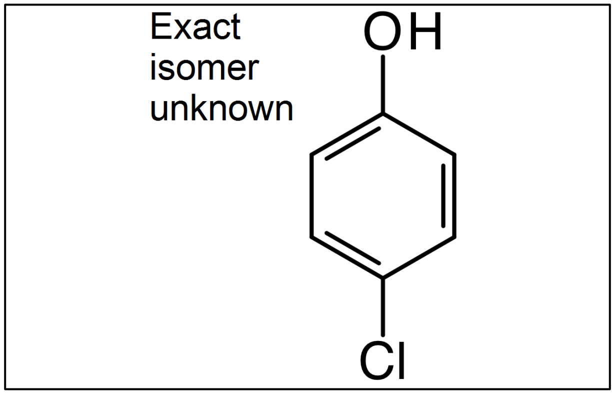

m/z [Fragment]
128 M+

Name: 4−Bromophenol

Class: Phenol N

Matrix: SD Bay Black Skimmer Egg
In Samples: All
Group: Not Typically Monitored
Comment:

Instrument: GCxGC−TOF, EI, 70 eV
1D RT, 2D RT (s): 1065.23, 1.386
Quantitative Ion m/z: 172

Elemental Formula: C6H5BrO
Source: Natural
Identification: Authentic MS RT

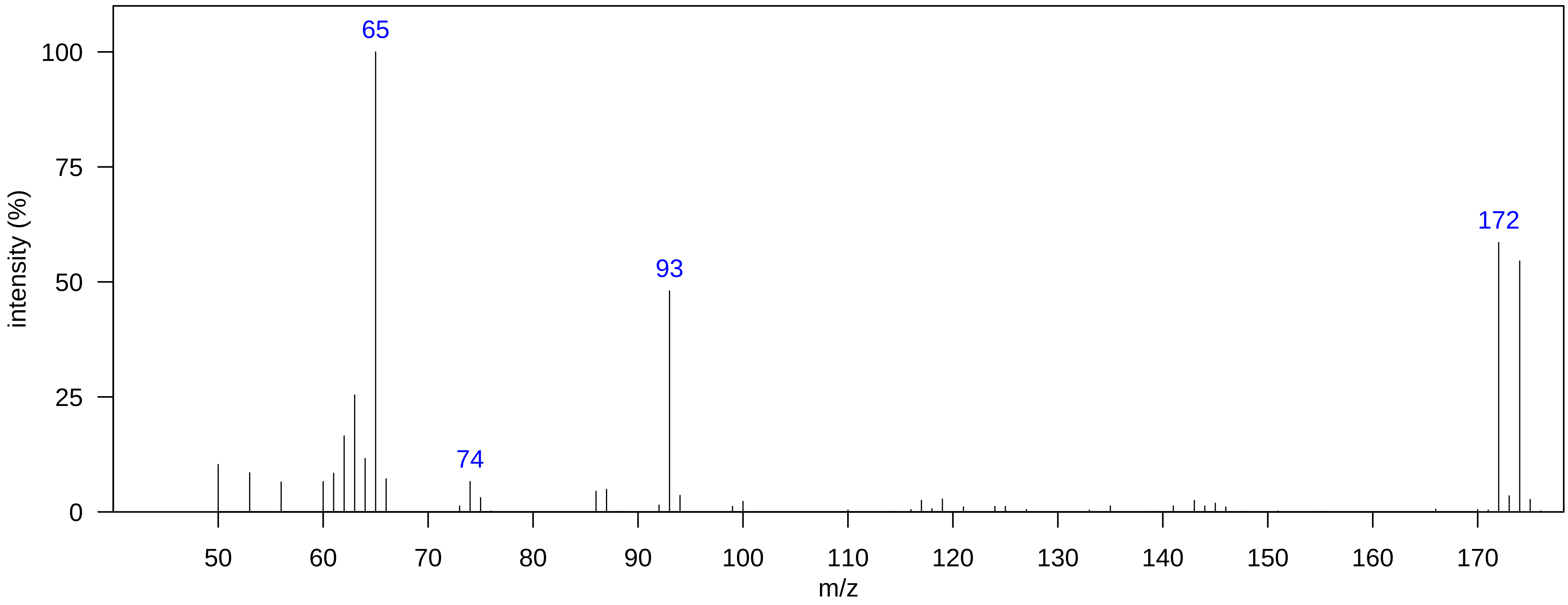

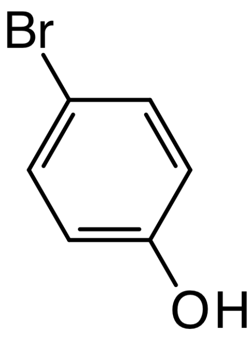

m/z [Fragment]
93 [M−Br]+
172 M+

Name: 4−Chlorocatechol

Class: Phenol A

Matrix: SD Bay Black Skimmer Egg
In Samples: A, B, C
Group: Not Typically Monitored
Comment:

Instrument: GCxGC−TOF, EI, 70 eV
1D RT, 2D RT (s): 1233.14, 1.287
Quantitative Ion m/z: 144

Elemental Formula: C6H5ClO2
Source: Anthropogenic
Identification: Authentic MS RT

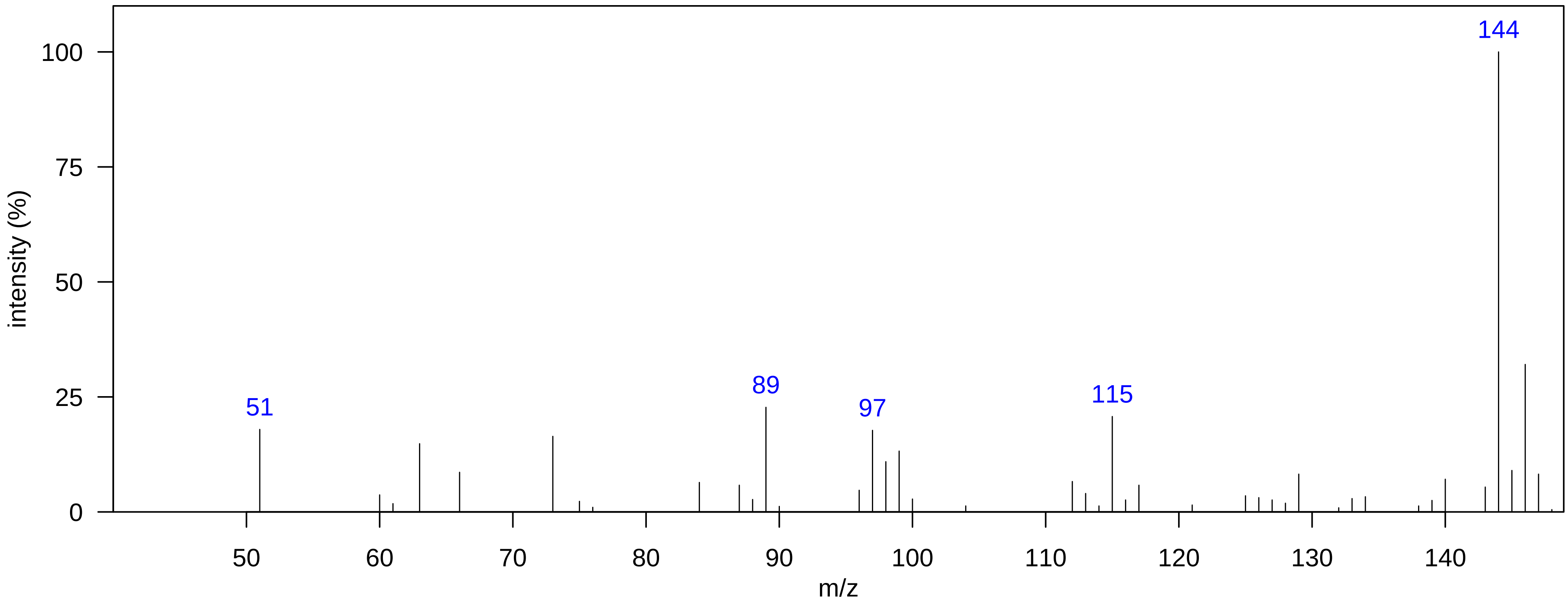

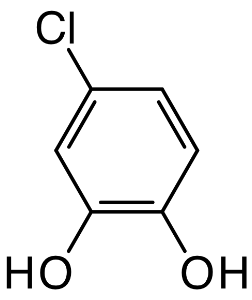

m/z [Fragment]
144 M+

Name: 4−Chlorocatechol isomer

Class: Phenol A

Matrix: SD Bay Black Skimmer Egg
In Samples: A, B, C
Group: Not Typically Monitored
Comment:

Instrument: GCxGC−TOF, EI, 70 eV
1D RT, 2D RT (s): 1355.57, 1.28
Quantitative Ion m/z: 144

Elemental Formula: C6H5ClO2
Source: Anthropogenic
Identification: Authentic MS

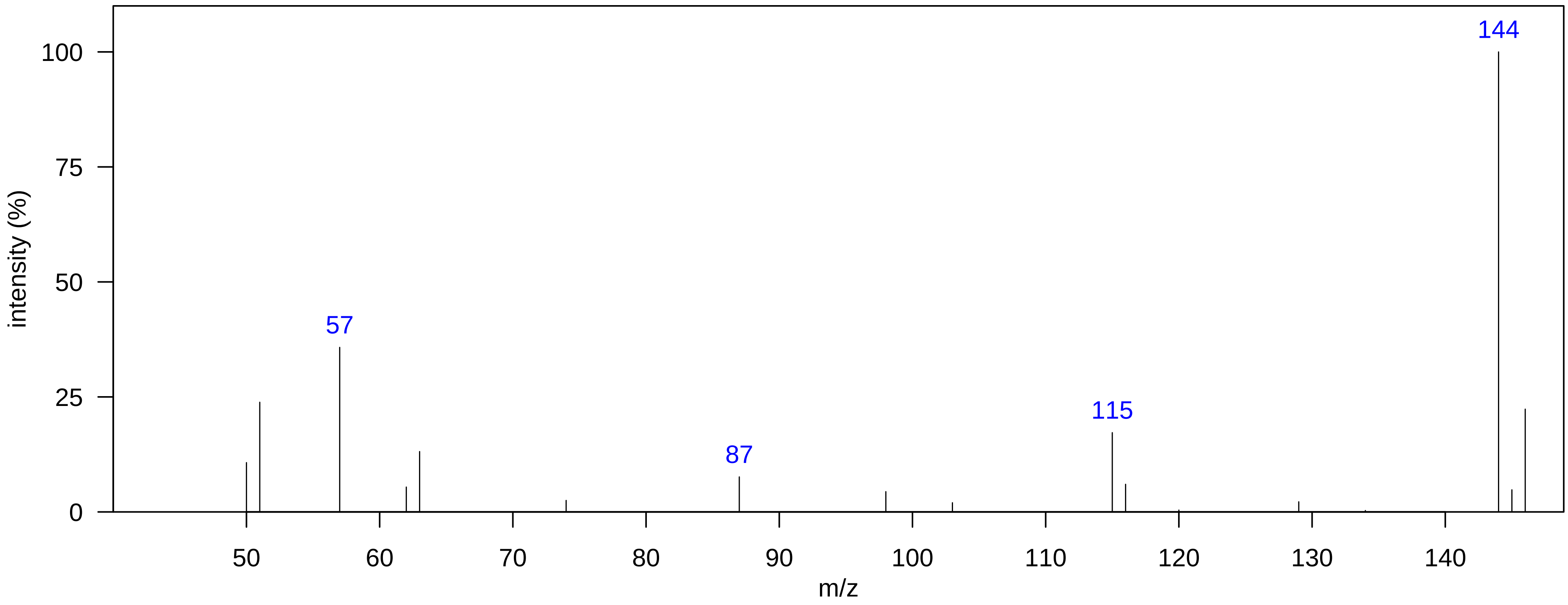

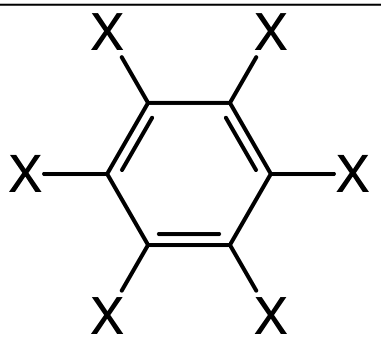

m/z [Fragment]
144 M+

Name: 2,4,6−Tribromoanisole

Class: Phenol M

Matrix: SD Bay Black Skimmer Egg
In Samples: A, C
Group: Not Typically Monitored
Comment:

Instrument: GCxGC−TOF, EI, 70 eV
1D RT, 2D RT (s): 1478, 1.465
Quantitative Ion m/z: 331

Elemental Formula: C7H5Br3O
Source: Mixed
Identification: Authentic MS RT

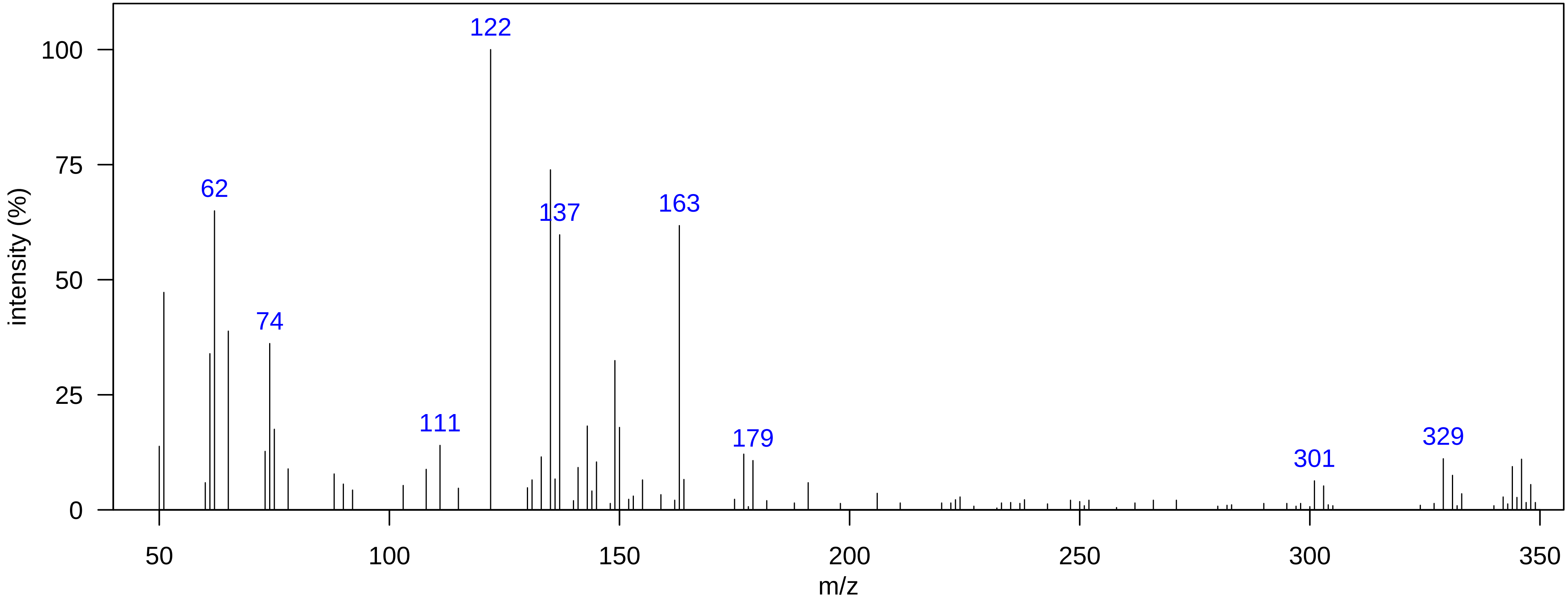

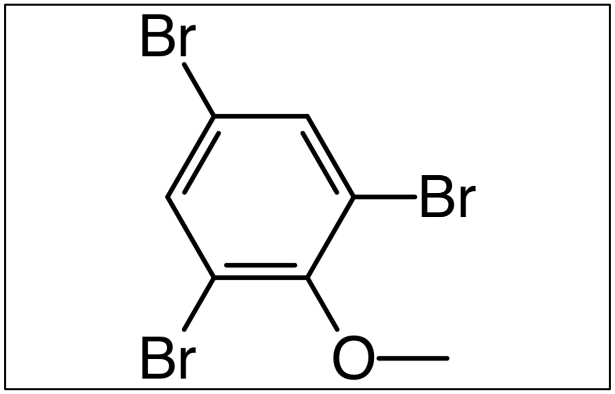

m/z [Fragment]
299 [M−C2H3O]+
327 [M−CH3]+
342 M+

Name: p−Chloroanisole

Class: Phenol A

Matrix: SD Bay Black Skimmer Egg
In Samples: A, C, D
Group: Not Typically Monitored
Comment: NIST I.D. is 1−Chloro−4−methoxybenzene.

Instrument: GCxGC−TOF, EI, 70 eV
1D RT, 2D RT (s): 1596.93, 1.082
Quantitative Ion m/z: 142

Elemental Formula: C7H7ClO
Source: Anthropogenic
Identification: Reference Database MS

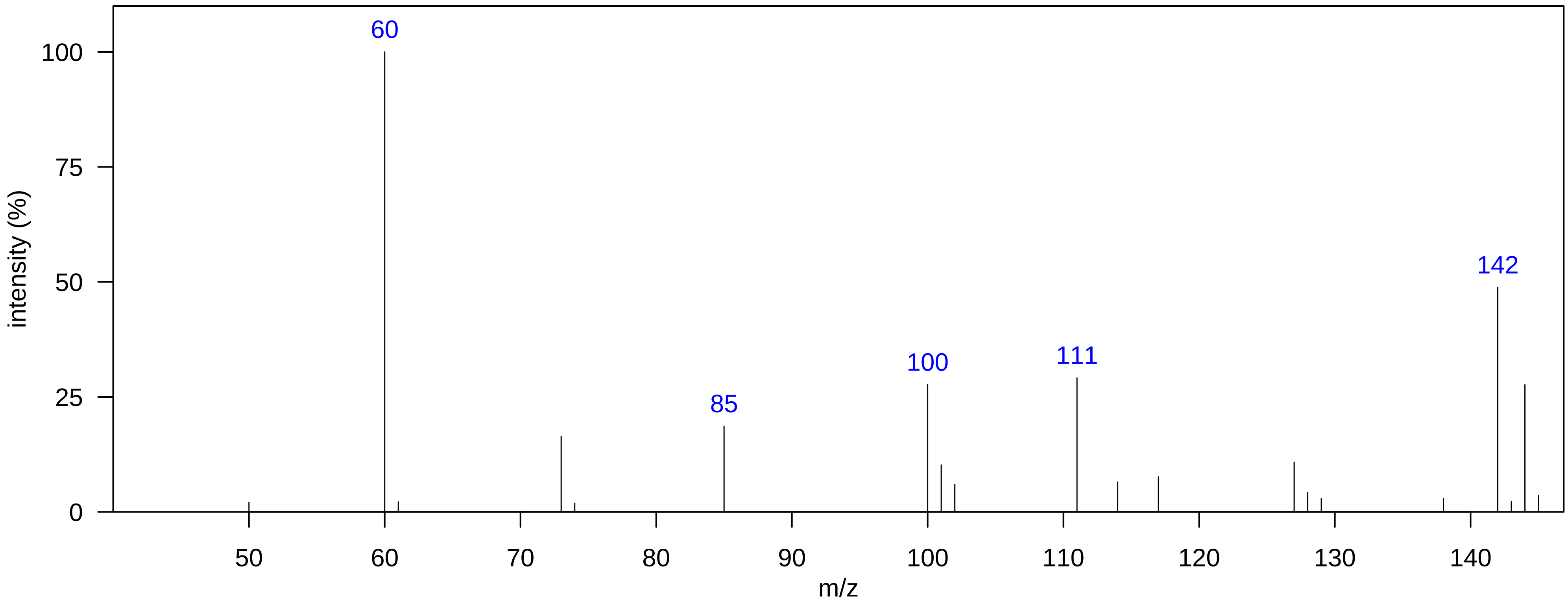

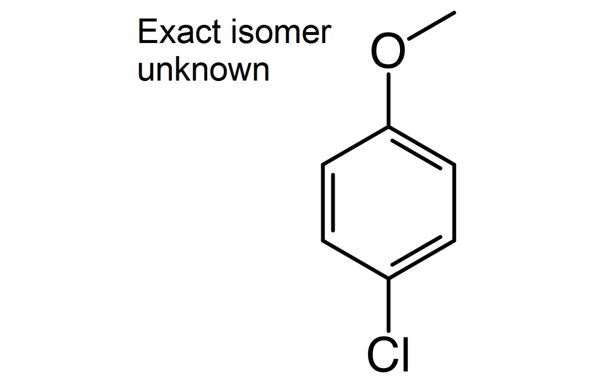

m/z [Fragment]
142 M+

Name: Triclosan

Class: Phenol A

Matrix: SD Bay Black Skimmer Egg
In Samples: All
Group: Not Typically Monitored
Comment:

Instrument: GCxGC−TOF, EI, 70 eV
1D RT, 2D RT (s): 1960.72, 1.61
Quantitative Ion m/z: 288

Elemental Formula: C12H7Cl3O2
Source: Anthropogenic
Identification: Authentic MS RT

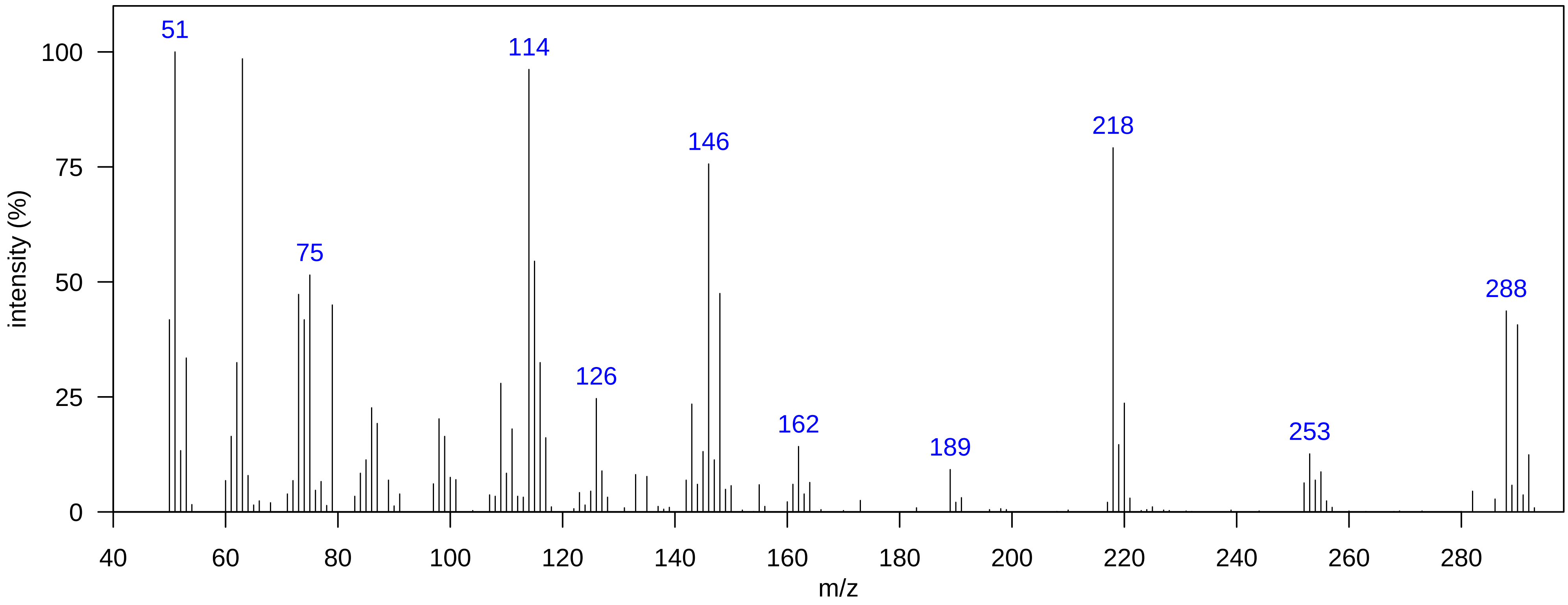

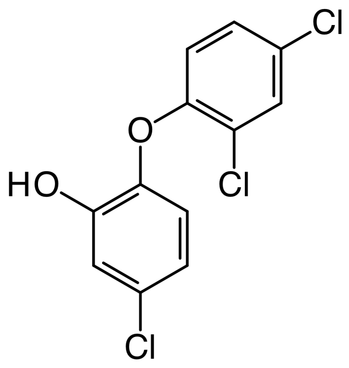

m/z [Fragment]
218 [M−Cl2]+
253 [M−Cl]+
288 M+

Name: Permethrin 1 Class: Pyrethroid

Matrix: SD Bay Black Skimmer Egg
In Samples: A, D
Group: Not Typically Monitored
Comment: M+ is not detected.

Instrument: GCxGC−TOF, EI, 70 eV
1D RT, 2D RT (s): 2408.47, 1.855
Quantitative Ion m/z: 183

Elemental Formula: C21H20Cl2O3
Source: Anthropogenic
Identification: Authentic MS RT

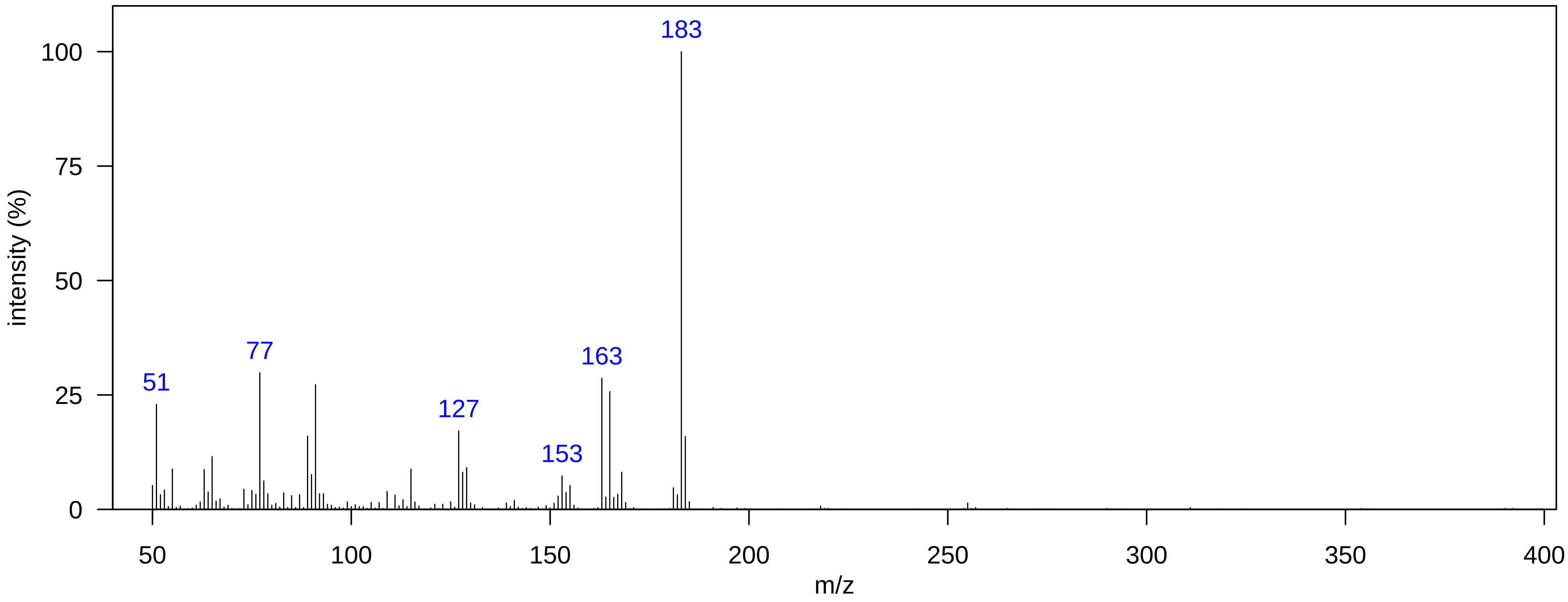

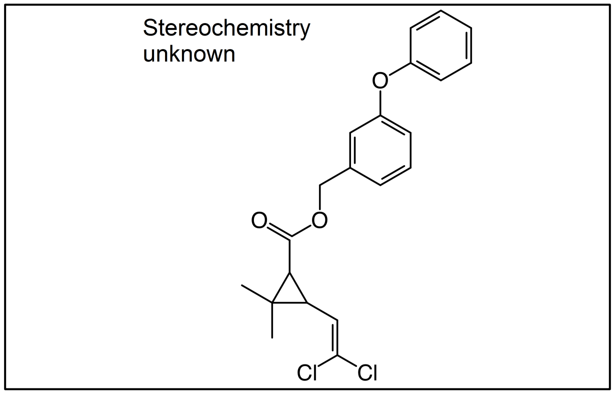

m/z [Fragment]
390 M+

Name: Permethrin 2

Class: Pyrethroid

Matrix: SD Bay Black Skimmer Egg
In Samples: A, D
Group: Not Typically Monitored
Comment: M+ is not detected.

Instrument: GCxGC−TOF, EI, 70 eV
1D RT, 2D RT (s): 2418.96, 1.868
Quantitative Ion m/z: 183

Elemental Formula: C21H20Cl2O3
Source: Anthropogenic
Identification: Authentic MS RT

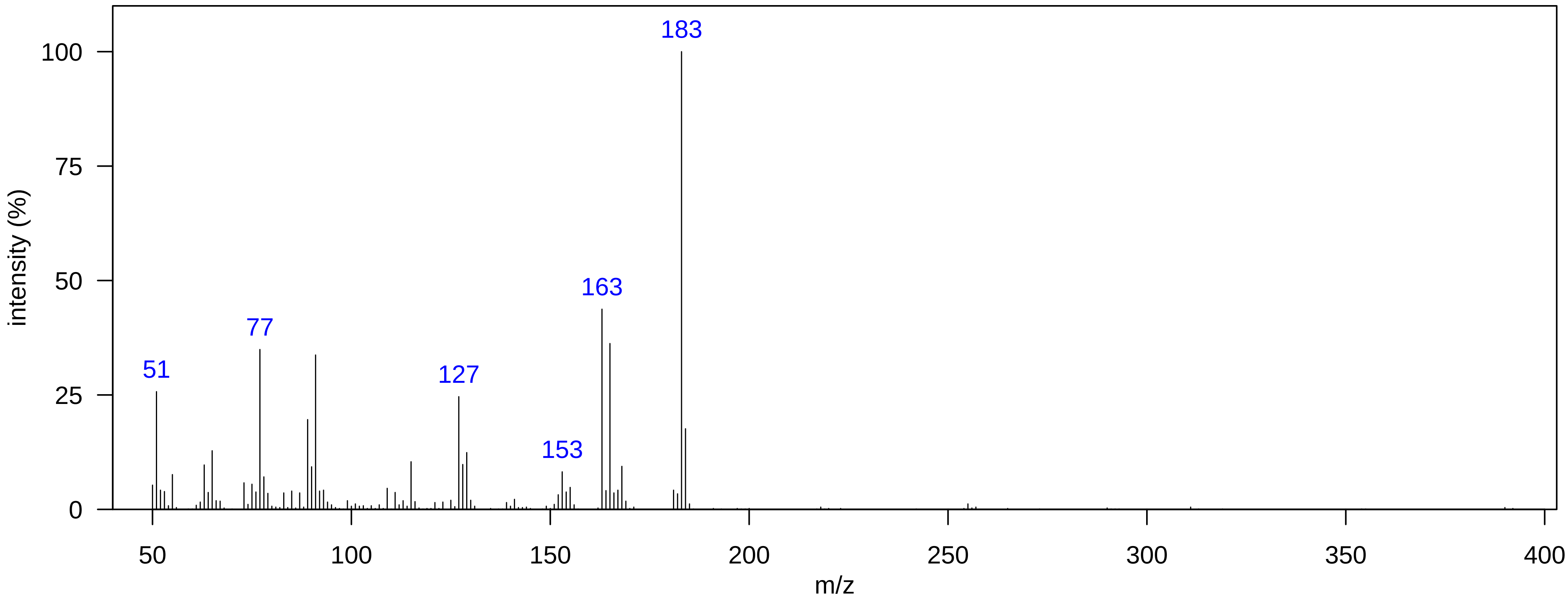

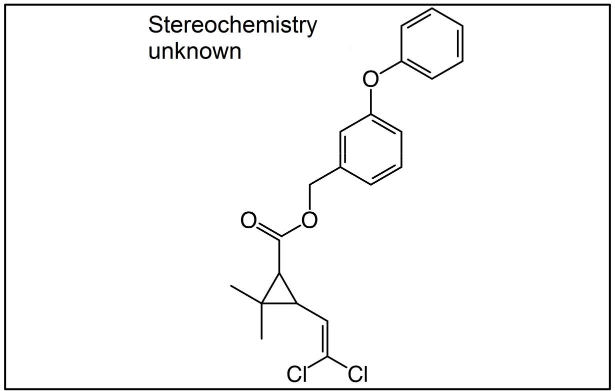

m/z [Fragment]
390 M+

Name: Cypermethrin

Class: Pyrethroid

Matrix: SD Bay Black Skimmer Egg
In Samples: A
Group: Not Typically Monitored
Comment: NIST I.D. is Cypermethrin. M+ is not detected.

Instrument: GCxGC−TOF, EI, 70 eV
1D RT, 2D RT (s): 2499.41, 1.987
Quantitative Ion m/z: 181

Elemental Formula: C22H19Cl2NO3
Source: Anthropogenic
Identification: Reference Database MS

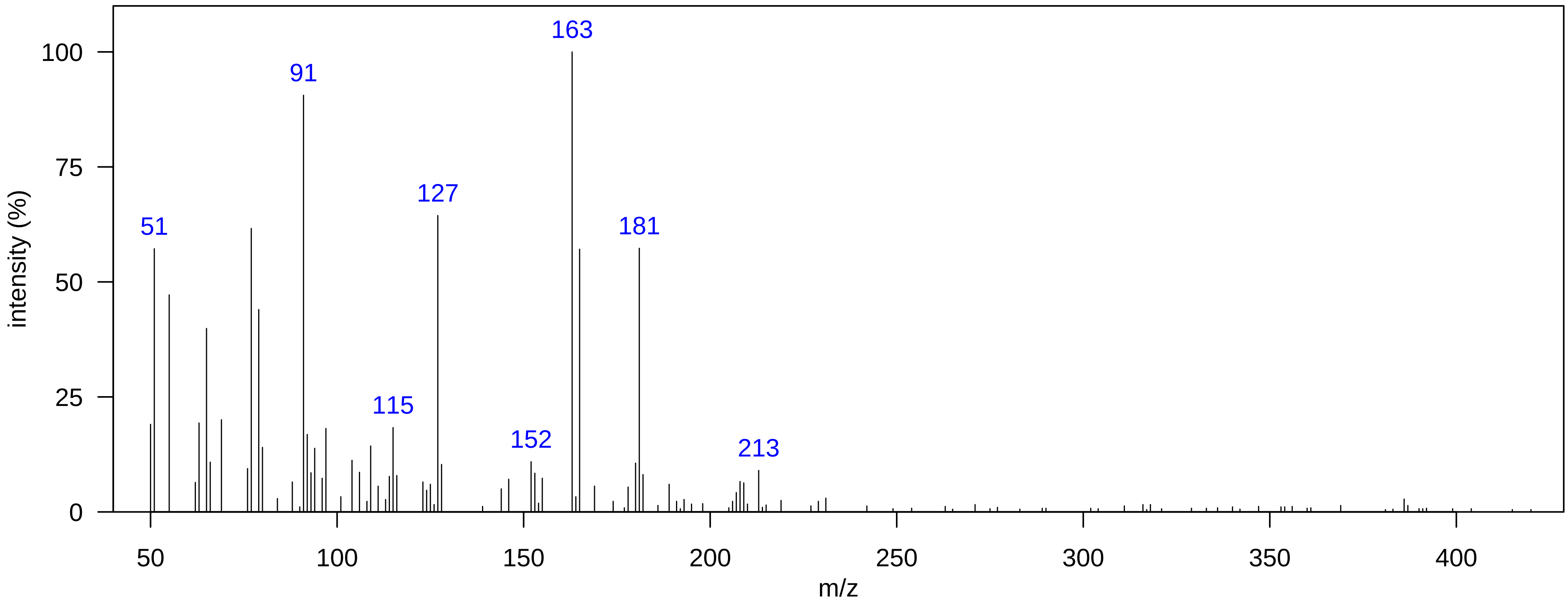

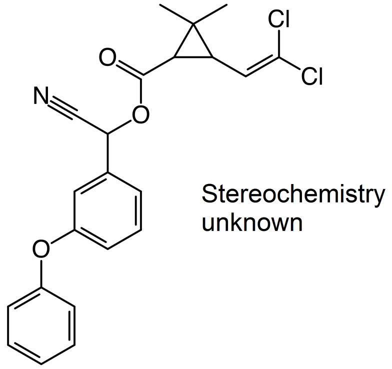

m/z [Fragment]
415 M+

Name: Unknown 1

Class: Unknown

Matrix: SD Bay Black Skimmer Egg
In Samples: All
Group: Not Typically Monitored
Comment:

Instrument: GCxGC−TOF, EI, 70 eV
1D RT, 2D RT (s): 1089.72, 1.339
Quantitative Ion m/z: 172

Elemental Formula:
Source: Unknown
Identification: Unknown

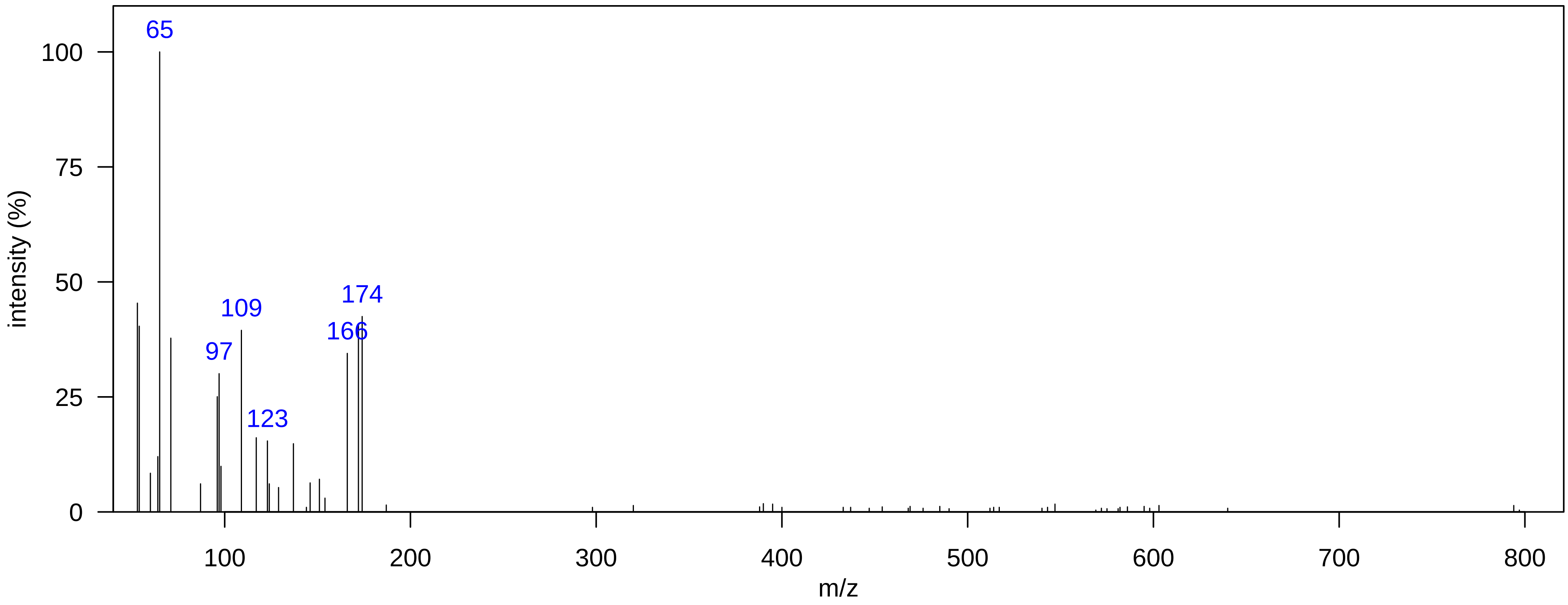

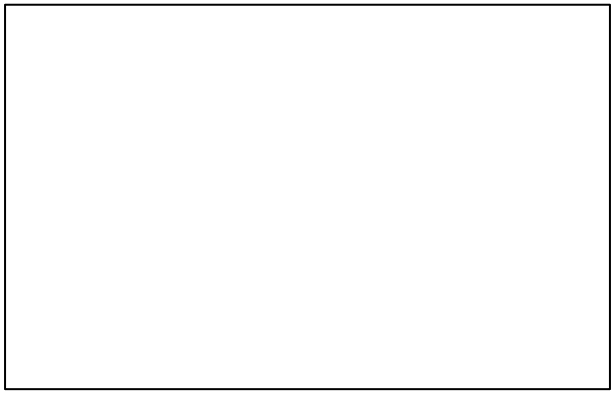

m/z [Fragment]

Name: Unknown 2A

Class: Unknown

Matrix: SD Bay Black Skimmer Egg
In Samples: All
Group: Not Typically Monitored
Comment:

Instrument: GCxGC−TOF, EI, 70 eV
1D RT, 2D RT (s): 1645.9, 1.445
Quantitative Ion m/z: 186

Elemental Formula:
Source: Unknown
Identification: Unknown

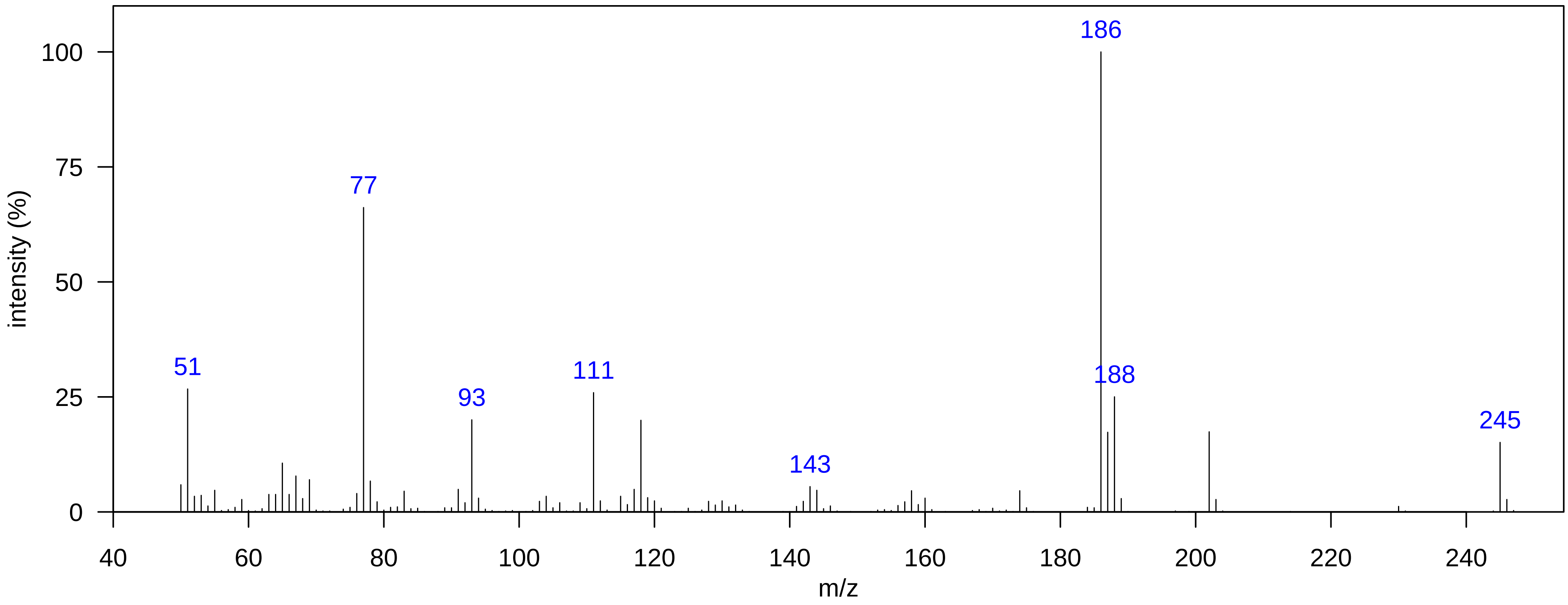

m/z [Fragment]

Name: Unknown 2B

Class: Unknown

Matrix: SD Bay Black Skimmer Egg
In Samples: All
Group: Not Typically Monitored
Comment:

Instrument: GCxGC−TOF, EI, 70 eV
1D RT, 2D RT (s): 1736.85, 1.432
Quantitative Ion m/z: 186

Elemental Formula:
Source: Unknown
Identification: Unknown

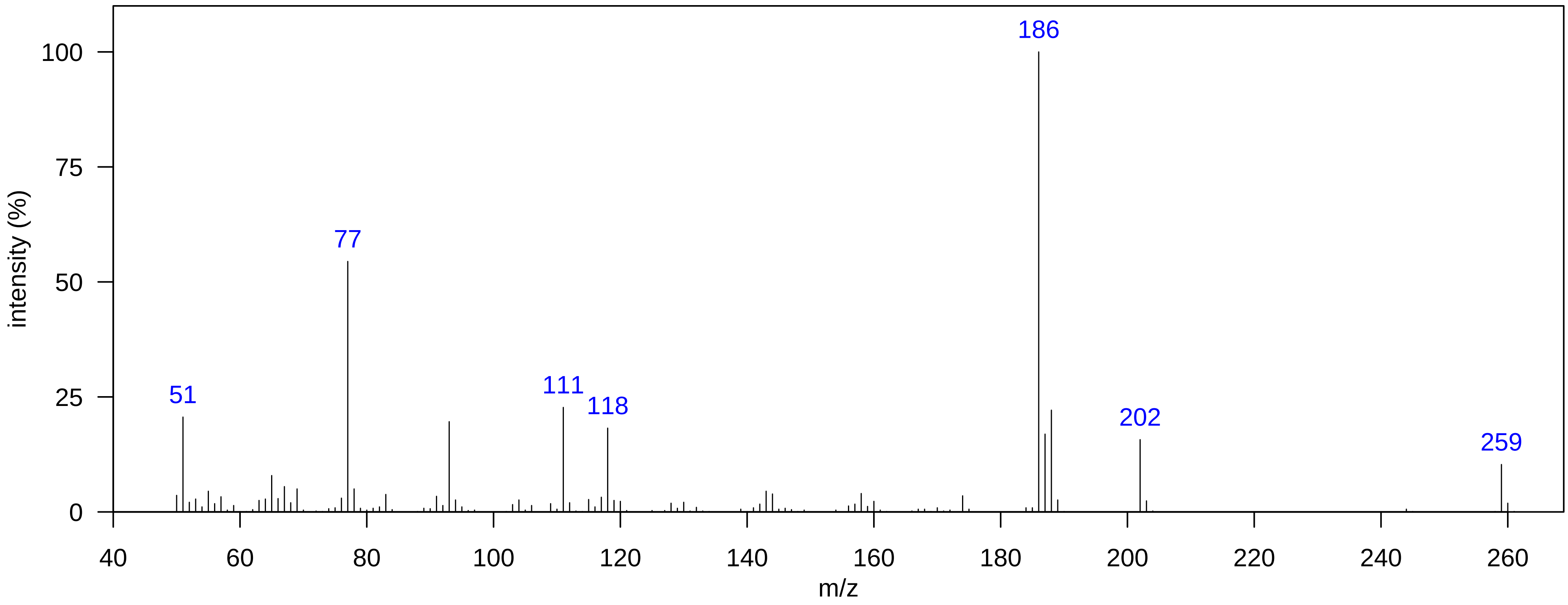

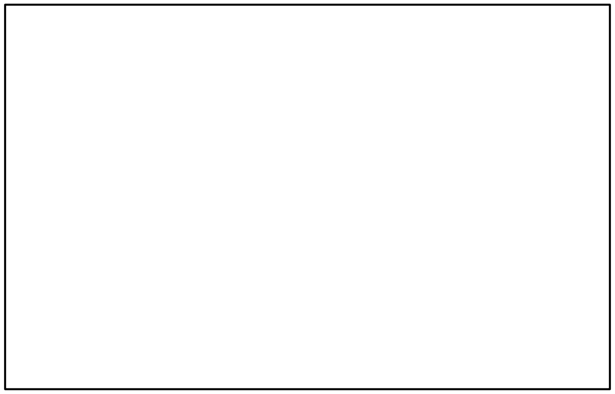

m/z [Fragment]

Name: Unknown 3 Class: Unknown

Matrix: SD Bay Black Skimmer Egg
In Samples: All
Group: Not Typically Monitored
Comment:

Instrument: GCxGC−TOF, EI, 70 eV
1D RT, 2D RT (s): 2167.1, 1.656
Quantitative Ion m/z: 282

Elemental Formula:
Source: Unknown
Identification: Unknown

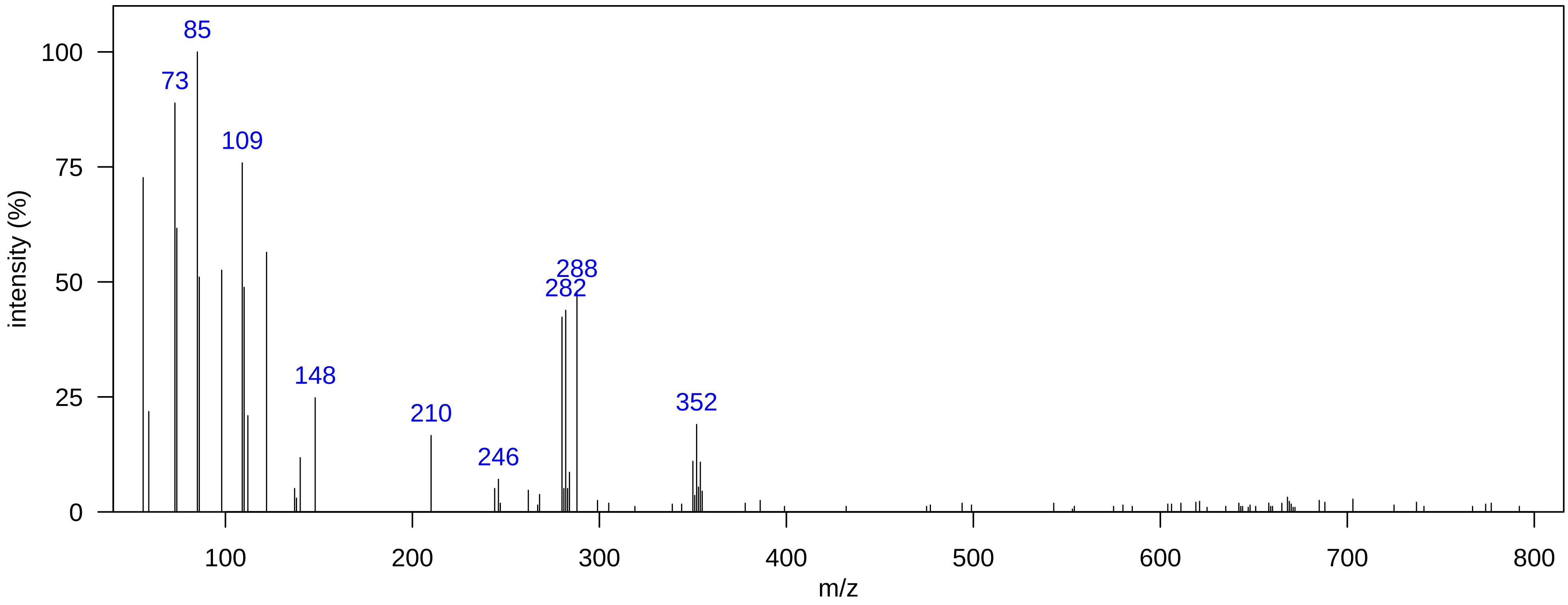

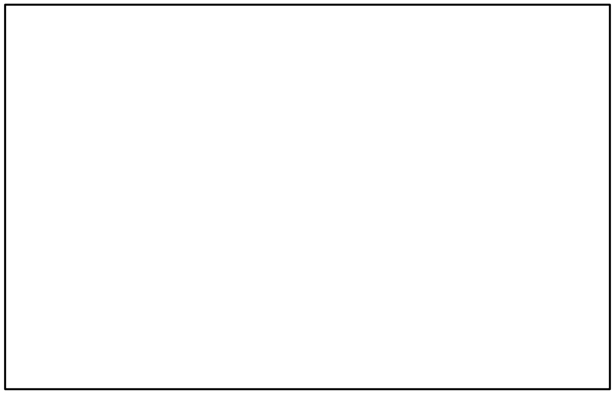

m/z [Fragment]
