# Supplementary material for: Identifying Bioaccumulative Halogenated Organic Compounds Using a Nontargeted Analytical Approach: Seabirds as Sentinels
Source: PLoS One. 2015 May 28;10(5):e0127205. doi: 10.1371/journal.pone.0127205 (PMC4447384; doi:10.1371/journal.pone.0127205)
Supplement: S2 File — (PDF) [file pone.0127205.s005.pdf]

Supporting Information

Identifying Bioaccumulative Halogenated Organic Compounds Using a Nontargeted Analytical Approach: Seabirds as Sentinels

Christopher J. Millow[1*], Susan A. Mackintosh[2,3], Rebecca L. Lewison[1], Nathan G. Dodder[4], and Eunha Hoh[2]

[1] Ecology Program Area, Department of Biology, San Diego State University, San Diego, California, United States of America

[2] Graduate School of Public Health, San Diego State University, San Diego, California, United States of America

[3] San Diego State University Research Foundation, San Diego, California, United States of America

[4] Southern California Coastal Water Research Project Authority, Costa Mesa, California, United States of America

* Corresponding author

Email: cmillow@gmail.com

Methods

Liquid-Liquid Extraction

For each sample, five grams of egg homogenate was transferred to a baked 50 mL glass Pyrex centrifuge tube and spiked with 50 µL of internal standards that included: 1 ng/µL of PCB mixture containing 2,3,5,6-tetrachlorobiphenyl, 2,2',4,4',6,6'-hexachlorobiphenyl, and 2,2',3,4,4',5,6,6'-octachlorobiphenyl (individually purchased from AccuStandard, New Haven, CT, USA) and 2 ng/µL of 4'-fluoro-2,3',4,6-tetrabromodiphenyl ether (FBDE-4001S; AccuStandard, New Haven, CT, USA). Prepared simultaneously was a reference blank sample (5g nano-pure LCMS grade water) spiked with 50 µL of internal standards. Blank samples were prepared and processed following the exact protocol of egg samples. Samples were then liquid-liquid extracted and purified to minimize bias and/or loss of broad chemical classes. First, 1 mL HCl 6N and 5 mL isopropanol was added to each sample, then vortexed for 3 minutes to denature proteins and ensure complete recovery of the sample. Each sample received 5 mL of 1:4 (v:v) dichloromethane (DCM):hexane mixture, was vortexed for 3 minutes, and centrifuged for 15 minutes at 3000 rpm at 20˚ C with an IEC Centra CL3R (Thermo Fisher Scientific, Fair Lawn, NJ, USA). The liquid organic top layer was collected and stored in a baked graduated test tube. An additional 5 ml of 1:4 (v:v) DCM:hexane mixture was added to the 50 ml Pyrex vial, mixed for 3 minutes and centrifuged a second time. The liquid top layer was added to the graduated cylinder, and total volume of sample extract was recorded.

Accounting for Lipids

In order to account for lipids within each sample, 1 ml of liquid-liquid extract was dispensed onto an empty, pre-weighed aluminum boat and evaporated for 24 hours in a fume hood. After 24 hours, the boat with the evaporated sample was re-weighed to determine lipid mass.

Sample Clean Up, Part I: Gel Permeation Chromatography (GPC)

Prior to Gel Permeation Chromatography (GPC), samples were nitrogen-evaporated to approximately 2 ml in a Zymark TurboVap (Caliper Life Sciences, Hopkinton, MA, USA) with a water bath set to 40˚ C. Each sample then received 5 ml of hexane and was nitrogen-evaporated a second time to approximately 2 ml. The extracted samples were cleaned by GPC (GPC; J2 Scientific, Columbia, MO, USA) in order to remove lipids. The method was optimized to ensure recovery of target analytes.

Each 2 ml evaporated sample was divided into two GPC baked glass vials (labeled “A” and “B”) in order to not overload the instrument with lipids. Each A and B sub-sample were treated identically. First, 2 ml of Ethyl Acetate was added to each sample vial, which was then nitrogen-evaporated to 1 ml. After, 1 ml of cyclohexane was added to each vial, and then vials were filled to 5 ml using a 1:1(v:v) Ethyl Acetate:Cyclohexane mixture. Each GPC vial was placed in the GPC Input Tray while

three corresponding large baked test tubes were placed in the Output Tray to collect the eluted fractions.

The GPC column has a 2 cm i.d. and a length of 22.5 cm and is packed with 24 g of BioBeads S-X3 in 1:1 ethyl acetate/cyclohexane. The flow rate was 5 ml/minute and the mobile phase was 1:1 ethyl acetate/cyclohexane. The eluting fraction between 10 and 22 minutes was collected and concentrated to 2 ml under a flow of nitrogen gas. All three fractions were then combined (6 ml total volume) and further concentrated to 2 ml. Final extracts were placed in baked 4 ml amber vials and stored at -20˚C until Solid Phase Extraction.

Sample Clean Up, Part II: Solid Phase Extraction (SPE)

Solid Phase Extraction (SPE) was utilized for further cleanup. For each sample, one silica SPE cartridge (ENVIRO CLEAN® Extraction Column, UCT, Bristol, PA, USA) was attached to a baked SPE glass syringe and was conditioned with 2.5 ml of hexane into a waste tube, which was removed after conditioning. The flow rate was set to approximately 1 drop per second via a vacuum manifold. Samples were first pipetted into the individual cartridge/syringe attachments and vacuumed, allowing sample extract to drip into baked collection test tubes below. Each sample was then eluted into separate collection tubes for three fractions in order to maximize extraction of contaminants from the column. All three fractions were examined but no compounds of interest were detected in fractions two and three. Between each fraction, the cartridges were sealed, the vacuum was turned off, and collection tubes were removed, covered, and replaced with new collection tubes before adding the next fraction. Fraction 1 was eluted with 4 ml of

hexane followed by 4 ml of 1:9 (v:v) hexane:dichloromethane (DCM); Fraction 2 was eluted with 4 ml of 100% DCM; Fraction 3 was eluted with 4 ml of acetone followed by 4 ml of methanol. Each fraction was then placed into the TurboVap at 40˚ C until reduced to approximately 1.5 ml. All evaporated samples were then transferred to 2 ml GC vials. Fraction 1 was evaporated to 200 µL, spiked with 50 µL of recovery standard containing 1 ng/µL of 3,3',4,4',5,5'-hexachloro[$^{13}C_{12}$]biphenyl (MBP 169; Wellington Laboratories, Guelph, Ontario, Canada), and 2 ng/µL of 4'-fluoro-2,3,3',4,5,6-hexabromodiphenyl ether (FBDE-6001S; AccuStandard, New Haven, CT, USA), then evaporated to 100 µL and transferred to insert vials placed inside sterilized 2 ml GC vials. No recovery standard was added nor did further evaporation occur with Fractions 2 and 3. Samples were sealed and stored at -20˚ C until analysis.

Instrumentation for Chemical Analysis

Egg samples were analyzed by Pegasus® 4D GC×GC-TOF-MS (LECO, St. Joseph, MI, USA) coupled to an Agilent Technologies 7890A Gas Chromatography System (Agilent Santa Clara, CA, USA) with splitless mode and with ultra-pure grade helium (99.995% Airgas Wes El Cajon, CA USA) as the carrier gas. One µl of each final extract was injected for analysis (S1 Table).

Quality Assurance & Control

All glassware utilized for the experiment was baked in a muffle furnace at 450°C for 6 hours. The plasma samples were kept in a -20°C walk in freezer. Samples were covered aluminum foil throughout the experiment to prevent contamination. In addition,

laboratory equipment was washed with acetone and hexane three times prior to experimental use. Each set of samples prepared for analysis was coupled with a laboratory procedural blank that was simultaneously processed in GC×GC/TOF-MS.

Reference Standards

The following reference standards were purchased from AccuStandard (New Haven, CT, USA): organochlorine pesticide mixture containing, β-BHC, heptachlor epoxide, γ-Chlordane, α-Chlordane, trans-Nonachlor, and cis-Nonachlor (M-680P); *p,p'*-DDMU (P-424S); PBDEs Standard Solution for Accuracy and Precision (BDE-AAP-A); PCB mix WHO/NIST/NOAA Congener Lis (CWNN); PCB Congener Mix for West Coast Fish Studies (C-WCFS); Triclosan (PCC-001S-10X); 4-Bromophenol (BP-004S); Chlorinated Phenolics Mix (M-1653C-R); chlordane (technical grade, APP-9-037); and DDT (technical grade, P-346N). The following reference standards were purchased from Wellington Laboratories (Guelph, Canada): Tris(4-chlorophenyl)methane (T4CPM); and Tris(4-chlorophenyl)methanol (T4CPME). A custom pesticide standard was purchased from Ultra Scientific (N. Kingstown, RI, USA) containing Mirex, *p,p'*-DDE, dieldrin and hexachlorobenzene. 2,4,6-tribromoanisole; 2-bromo-1,3-diphenylpropane-1,3-dione; and Bis (4-Chlorophenyl) sulfone were purchased from Sigma-Aldrich (Milwaukee, WI, USA). Halogenated 1'-methyl-1,2'-bipyrrole (Q1, MBP-Cl7) was donated by Walter Vetter. A Pyrethrins and Pyrethroids mix was purchased from Chem Service (West Chester, PA, USA) (PP1660-2JM). Methylmercury(II) iodine standard was purchased from Alfra Aesar (Ward Hill, MA, USA) (A18340).

Figure Legends

S1 Fig. Relative Abundance of Individual Compounds. Each point represents one egg sample (n=4), including non-detects. Non-detects are shown with a value of zero (multiple non-detects overlap).

Tables

S1 Table. GC×GC/TOF-MS Analysis Conditions.

S2 Table. Complete Compound Detection Frequency Per Egg.
